# Supplementary material for: Effectiveness, cost-effectiveness and safety of gabapentin versus placebo as an adjunct to multimodal pain regimens in surgical patients: protocol of a placebo controlled randomised controlled trial with blinding (GAP study)
Source: BMJ Open. 2020 Nov 20;10(11):e041176. doi: 10.1136/bmjopen-2020-041176 (PMC7682449; doi:10.1136/bmjopen-2020-041176)
Supplement: Author's manuscript [file bmjopen-2020-041176.draft_revisions.pdf]

BMJ Open is committed to open peer review. As part of this commitment we make the peer review history of every article we publish publicly available.

When an article is published we post the peer reviewers' comments and the authors' responses online. We also post the versions of the paper that were used during peer review. These are the versions that the peer review comments apply to.

The versions of the paper that follow are the versions that were submitted during the peer review process. They are not the versions of record or the final published versions. They should not be cited or distributed as the published version of this manuscript.

BMJ Open is an open access journal and the full, final, typeset and author-corrected version of record of the manuscript is available on our site with no access controls, subscription charges or pay-per-view fees (http://bmjopen.bmj.com).

If you have any questions on BMJ Open's open peer review process please email info.bmjopen@bmj.com

BMJ Open

Effectiveness, cost effectiveness and safety of gabapentin versus placebo as an adjunct to multimodal pain regimens in surgical patients: Protocol of a placebo controlled randomised controlled trial with blinding (GAP study)

Journal:	*BMJ Open*
Manuscript ID	bmjopen-2020-041176
Article Type:	Protocol
Date Submitted by the Author:	02-Jun-2020

Complete List of Authors:	Baos, Sarah; University of Bristol, Bristol Trials Centre, Clinical Trials and Evaluation Unit<br>Rogers, Chris; University of Bristol, Bristol Trials Centre, Clinical Trials and Evaluation Unit<br>Abbadi, Reyad; University Hospitals Bristol and Weston NHS Foundation Trust<br>Alzetani, Aiman; University Hospital Southampton NHS Foundation Trust<br>Casali, Gianluca; University Hospitals Bristol and Weston NHS Foundation Trust<br>Chauhan, Nilesh; University of Bristol and Weston NHS Foundation Trust<br>Collett, Laura; University of Bristol, Bristol Trials Centre, Clinical Trials and Evaluation Unit<br>Culliford, Lucy; University of Bristol, Bristol Trials Centre, Clinical Trials and Evaluation Unit<br>de Jesus, Samantha; University of Bristol, Bristol Trials Centre, Clinical Trials and Evaluation Unit<br>Edwards, Mark; University Hospital Southampton NHS Foundation Trust, Department of Anaesthesia; NIHR Biomedical Research Centre, University Southampton NHS Foundation Trust and University of Southampton, Acute, Critical & Perioperative Care Research Group<br>Goddard, Nicholas; University Hospital Southampton NHS Foundation Trust<br>Lamb, Jennifer; University of Bristol, Bristol Trials Centre, Clinical Trials and Evaluation Unit<br>McKeon, Holly; University of Bristol, Bristol Trials Centre, Clinical Trials and Evaluation Unit<br>Molyneux, Mat; University of Bristol and Weston NHS Foundation Trust<br>Stokes, Elizabeth; University of Oxford, Health Economics Research Centre Nuffield Department of Population Health<br>Wordsworth, Sarah; University of Oxford, University of Oxford<br>Gibbison, Ben; University of Bristol and Weston NHS Foundation Trust; University of Bristol<br>Pufulete, Maria; University of Bristol, Bristol Trials Centre, Clinical Trials and Evaluation Unit

Keywords:	Clinical trials < THERAPEUTICS, Pain management < ANAESTHETICS, SURGERY

SCHOLARONE™
Manuscripts

I, the Submitting Author has the right to grant and does grant on behalf of all authors of the Work (as defined in the below author licence), an exclusive licence and/or a non-exclusive licence for contributions from authors who are: i) UK Crown employees; ii) where BMJ has agreed a CC-BY licence shall apply, and/or iii) in accordance with the terms applicable for US Federal Government officers or employees acting as part of their official duties; on a worldwide, perpetual, irrevocable, royalty-free basis to BMJ Publishing Group Ltd ("BMJ") its licensees and where the relevant Journal is co-owned by BMJ to the co-owners of the Journal, to publish the Work in this journal and any other BMJ products and to exploit all rights, as set out in our licence.

The Submitting Author accepts and understands that any supply made under these terms is made by BMJ to the Submitting Author unless you are acting as an employee on behalf of your employer or a postgraduate student of an affiliated institution which is paying any applicable article publishing charge ("APC") for Open Access articles. Where the Submitting Author wishes to make the Work available on an Open Access basis (and intends to pay the relevant APC), the terms of reuse of such Open Access shall be governed by a Creative Commons licence – details of these licences and which Creative Commons licence will apply to this Work are set out in our licence referred to above.

Other than as permitted in any relevant BMJ Author's Self Archiving Policies, I confirm this Work has not been accepted for publication elsewhere, is not being considered for publication elsewhere and does not duplicate material already published. I confirm all authors consent to publication of this Work and authorise the granting of this licence.

For peer review only

Effectiveness, cost effectiveness and safety of gabapentin versus placebo as an adjunct to multimodal pain regimens in surgical patients: Protocol of a placebo controlled randomised controlled trial with blinding (GAP study)

Sarah Baos[1] sarah.baos@bristol.ac.uk

Chris A Rogers[1] chris.rogers@bristol.ac.uk

Reyad Al-Ghnaniem Abbadi[2] reyad.abbadi@UHBristol.nhs.uk

Aiman Alzetani[3] aiman.alzetani@uhs.nhs.uk

Gianluca Casali[2] gcasali@its.jnj.com

Nilesh Chauhan[2] nilesh.chauhan@uhbristol.nhs.uk

Laura Collett[1] laura.collett@bristol.ac.uk

Lucy Culliford[1] lucy.culliford@bristol.ac.uk

Samantha E de Jesus[1] samantha.dejesus@bristol.ac.uk

Mark Edwards[4, 5] mark.edwards2@uhs.nhs.uk

Nick Goddard[3] nicholas.goddard@uhs.nhs.uk

Jennifer Lamb[1] jenny.lamb@bristol.ac.uk

Holly McKeon[1] holly.mckeon@bristol.ac.uk

Mat Molyneux[2] mat.molyneux@uhbristol.nhs.uk

Elizabeth Stokes[6] elizabeth.stokes@dph.ox.ac.uk

Sarah Wordsworth[6] sarah.wordsworth@dph.ox.ac.uk

Ben Gibbison[2, 7] ben.gibbison@bristol.ac.uk

Maria Pufulete[1] maria.pufulete@bristol.ac.uk

For peer review only - http://bmjopen.bmj.com/site/about/guidelines.xhtml

1. Bristol Trials Centre, Clinical Trials and Evaluation Unit, University of Bristol, Bristol, UK

2. University Hospitals Bristol and Weston NHS Foundation Trust, Bristol, UK

3. University Hospital Southampton NHS Foundation Trust, Southampton, UK

4. Department of Anaesthesia, University Hospital Southampton NHS Foundation Trust, Southampton, UK

5. Acute, Critical & Perioperative Care Research Group, NIHR Biomedical Research Centre, University Hospital Southampton NHS Foundation Trust / University of Southampton, Southampton, UK

6. University of Oxford, Oxford, UK

7. University of Bristol, Bristol, UK

Corresponding author: Sarah Baos, Bristol Trials Centre, Clinical Trials and Evaluation Unit, University of Bristol, Bristol, UK. Email: sarah.baos@bristol.ac.uk. Tel: 0117 342 2297

Word count: 3998 (excluding title page, abstract, figures and tables, acknowledgements, contributions and references)

Number of figures: 1

Number of tables: 1

Keywords: Randomised controlled trial (RCT); gabapentin; placebo; pain management; peri-operative; surgical patients

For peer review only

ABSTRACT

Introduction: Gabapentin is an antiepileptic drug currently licensed to treat epilepsy and neuropathic pain but has been used off-label to treat acute post-operative pain. The GAP study will compare the effectiveness, cost-effectiveness and safety of gabapentin as an adjunct to standard multimodal analgesia versus placebo for the management of pain after major surgery.

Methods and analysis: The GAP study is a multi-centre, double-blind, randomised controlled trial in patients aged 18 years and over, undergoing different types of major surgery (cardiac, thoracic or abdominal). Patients will be randomised in a 1:1 ratio to receive either gabapentin (600mg just before surgery and 600mg/day for 2 days after surgery) or placebo in addition to usual pain management for each type of surgery. Patients will be followed up daily until hospital discharge and then at 4 weeks and 4 months after surgery. The primary outcome is length of hospital stay following surgery. Secondary outcomes include pain, total opioid use, adverse health events, health related quality of life and costs.

Ethics and dissemination: This study has been approved by the National Research Ethics Service. Findings will be shared with participating hospitals and disseminated to the academic community through peer reviewed publications and presentation at national and international meetings. Patients will be informed of the results through patient organisations and participant newsletters.

Trial registration: ISRCTN63614165. Registered on 05/06/2017.

For peer review only - http://bmjopen.bmj.com/site/about/guidelines.xhtml

ARTICLE SUMMARY

Strengths and limitations of this study

- Pragmatic design integrated into standard care pathways
- First trial to assess the impact of gabapentin on hospital stay and quality of life
- Implemented in three types of major surgery: cardiac, thoracic and abdominal
- Non-variable dose and limited duration of intervention may reduce applicability (e.g. frail / infirm patients and patients requiring analgesia for longer than 2 days)
- Only includes major body cavity surgery, which reduces applicability to major orthopaedic surgery

INTRODUCTION

About 4.7 million patients undergo surgery in the UK each year (1). Many patients experience significant pain after surgery and about 10% experience severe pain (2-5). Inadequate pain management increases the length of hospital stay (6) and contributes to the development of chronic or persistent post-surgical pain (7, 8), which impacts on quality of life (9). Current multimodal analgesic regimens include paracetamol, non-steroidal anti-inflammatory drugs (NSAIDs), regional analgesia (focused delivery of a local anaesthetic to a specific part of the body) and opioids.

Opioids are key analgesic agents for managing moderate to severe pain. However, they have poor efficacy in movement-associated pain and have significant side-effects including confusion, nausea, vomiting, itching, constipation and respiratory depression. These side effects can increase the length of hospital stay, delay overall recovery and reduce quality of life (9). Reliance on opioids after surgery also increases the risk of long-term use and opioid dependence (10, 11). Gabapentin is

For peer review only

an anti-epileptic drug currently licensed to treat epileptic convulsions and neuropathic pain. It is also commonly used off-label in the peri-operative setting to reduce opioid use without compromising pain control. We conducted a survey of UK practice amongst consultant anaesthetists in the South West of England and members of the British Pain Society Acute Pain Special Interest Group. We found that 35/145 (23%) of anaesthetists prescribe gabapentin to their patients, with large variation in practice across the UK (12). Reducing opioid use after surgery to return patients to full health as quickly as possible, is one of the central tenets of enhanced recovery in the NHS (13). However, there is currently no robust evidence to recommend the inclusion of gabapentin in enhanced recovery protocols.

There are over 130 randomised controlled trials (RCTs) that have investigated gabapentin versus placebo in different surgical populations. Most of these trials are small (<200 patients, median 80) and highly heterogeneous, both statistically and clinically. These RCTs have been included in 17 systematic reviews that aimed to assess the effectiveness of gabapentin versus placebo in the peri-operative period; 10 of these in surgical populations (14-23). All reviews reached the same conclusions; that gabapentin reduced opioid consumption and post-operative pain scores at 24 hours ($P<0.001$), but none has assessed the impact on length of hospital stay or quality of life.

The GAP study will compare the safety, effectiveness and cost-effectiveness of gabapentin as an adjunct to standard multimodal analgesia versus placebo for the management of pain after surgery. Specific objectives are to estimate: i) the difference between groups in length of hospital stay following surgery; ii) the

For peer review only - http://bmjopen.bmj.com/site/about/guidelines.xhtml

difference between groups in total opioid use, pain, adverse events and health-related quality of life (HRQoL); and iii) the cost effectiveness of gabapentin compared to usual care.

METHODS AND ANALYSIS

Trial design and population

The GAP study is a multi-centre, parallel group, placebo-controlled, pragmatic double-blind RCT. Patients will be recruited from three surgical specialties (cardiac, thoracic and abdominal) across several secondary care NHS centres (Figure 1). A principal investigator will be appointed in each centre and clinical leads will be identified for each specialty within each centre.

GAP includes two phases: i) Phase 1 (12 months) involves study set-up and recruitment from two NHS secondary centres (University Hospitals Bristol and Weston NHS Foundation Trust and University Hospitals Southampton NHS Foundation Trust) with integrated monitoring of the recruitment process to maximise recruitment and adherence with the study medication; ii) Phase 2 (18 months) continued recruitment using the optimum methods established in phase 1, opening additional centres (if required). Progression from phase 1 to phase 2 is contingent on demonstrating that after 9 months of recruitment in phase 1, sufficient numbers of patients referred for surgery are eligible for the trial and can be enrolled to complete the main trial. Specifically:

1. At least 60% of patients undergoing surgery are considered eligible;
2. At least 50% of eligible patients consent to randomisation by 6 months of recruitment at each centre.

For peer review only - http://bmjopen.bmj.com/site/about/guidelines.xhtml

Eligibility criteria

Patients will be eligible for the study if all the following apply:

1. Over 18 years of age;
2. Undergoing non-emergency surgery:

   i) Cardiac (surgery on the heart and great vessels performed via midline sternotomy);

   ii) Thoracic (open or minimal access surgery on the lungs and surrounding tissues);

   iii) Abdominal (open or minimal access surgery within the abdominal cavity);
3. Expected to stay in hospital at least until day 2 after surgery (day 0 is day of surgery);
4. Expected to be able to swallow during the time of the study intervention.

Patients will be excluded from the study if any of the following apply:

1. Taking anti-epileptic medication(s);
2. Gabapentin allergy;
3. Already taking gabapentin or gabapentanoids;
4. Galactose intolerance, Lapp lactase deficiency or glucose-galactose malabsorption;
5. Planned epidural analgesia;
6. Intended use of any gabapentanoids in the peri-operative analgesic protocol other than the study medication (this includes but is not restricted to: pregabalin, enacarbil gabapentin, 4-methylpregabalin and phenibut);
7. Known renal impairment (estimated glomerular filtration rate (eGFR) $<30ml/min/1.73^2$);
8. Weight <50kg;

For peer review only - http://bmjopen.bmj.com/site/about/guidelines.xhtml

9. Inability to provide written informed consent;
10. Unwilling to participate in follow-up;
11. Prisoners;
12. Enrolled in another clinical trial and: i) the patient is currently taking an investigational medicinal product as part of the other trial; or ii) co-enrolment is not permitted by the other trial; or iii) co-enrolment would be burdensome for the patient.

Patient approach and consent

Potential patients will be identified from clinic and planned operating lists and those eligible to participate will receive a patient information leaflet (PIL). Most patients will have at least 24 hours to consider participation. However, it is important to include urgent, non-emergency patients who may have less than 24 hours to consider the study, to maximise the applicability of the findings. In these circumstances, patients will only be enrolled if they confirm that they have had enough time to consider their participation.

Prior to surgery, patients will be seen by a member of the local research team who will answer any questions, confirm eligibility and receive written informed consent if the patient decides to participate. Details of all patients approached and reasons for non-participation (e.g. ineligibility or patient refusal) will be documented. The patients' General Practitioners will be informed of their enrolment in the study. Participants can withdraw at any time and will be treated according to standard hospital procedures. If a participant decides that they no longer wish to take part in study procedures, data collection for those procedures will cease. These participants

For peer review only - http://bmjopen.bmj.com/site/about/guidelines.xhtml

will be asked whether they are still willing to participate in the study follow-up, if applicable.

Interventions

The study intervention is gabapentin 600 mg given preoperatively and 600 mg/day (300 mg in the morning and 300 mg in the evening) given postoperatively for 2 days when clinically able to swallow following extubation (if applicable). The control is a placebo, taken at the same time-points as the active tablet. Both gabapentin and placebo will be administered within local multimodal analgesic regimens. The study medication (gabapentin/placebo) is manufactured, packaged and labelled in accordance with Good Manufacturing Practice and is stored at room temperature, below 25ºC.

Use of any gabapentanoids other than the study medication during the study intervention period is prohibited. If the pre-operative dose is administered and surgery is postponed by more than 12 hours, a second pre-operative dose of study medication will be given before the re-scheduled surgery. If a post-operative dose of study medication is missed by less than 6 hours, patients should be given the missed dose and continue to the next scheduled dose as per the protocol. If a dose of study medication is missed by 6 hours or more, patients should continue to the next scheduled dose and should not be given the missed dose. For patients intubated for longer than 48 hours after the end of surgery, no post-operative study medication should be administered. All other aspects of patient's care will be performed according to local practice.

For peer review only - http://bmjopen.bmj.com/site/about/guidelines.xhtml

Randomisation

Randomisation will be performed after eligibility has been confirmed, using a secure internet-based randomisation system to ensure allocation concealment. Patients will be allocated in a 1:1 ratio to either gabapentin or placebo. A computer-generated allocation sequence will be prepared by an the unblinded study statistician. The random allocation will be blocked with blocks of varying size and stratified by centre and specialty, so that each specialty at each centre will have approximately equal numbers of patients allocated to placebo and gabapentin. To maintain blinding, the randomisation system will only reveal a unique pack number, which identifies the study medication to be given.

Blinding

Patients, their clinical care team (i.e. surgeon, anaesthetist, and those responsible for their post-operative care) and the research nurses will not be informed of the allocation. Patients will be made aware before entering the study that they will not be told which treatment they will receive. Doctors will prescribe 'study medication' rather than specifically gabapentin or placebo. The unique pack number provided by the randomisation system will provide the medication as specified by the pre-determined randomisation list. The allocations will only be known by pharmacy and the unblinded study statistician and will not be disclosed to other members of the research team. The treatment allocation will only be unblinded if clinically indicated; for example, in the event of a suspected serious adverse reaction to the study medication, the management of which might be altered by knowledge of the allocation.

For peer review only - http://bmjopen.bmj.com/site/about/guidelines.xhtml

The study medication is over-encapsulated to maintain blinding. The capsules for active drug and placebo will look identical and do not have a particularly strong or unusual smell or taste, so we do not anticipate unblinding will occur due to the characteristics of the medication. Gabapentin may induce side-effects that may inadvertently unblind patients and/or clinical teams. However, given that the side effects of gabapentin (e.g. drowsiness, dizziness and difficulty concentrating) are similar to those of opioids, and that patients/clinical care teams are likely to view side effects as resulting from their whole surgical and post-operative experience, it is unlikely that any patient/clinician will definitively be able to attribute a specific side effect to gabapentin. The PIL and the process of informed consent explain the uncertainty around the potential beneficial effects of gabapentin over a placebo. Therefore, in the event of inadvertent unblinding, patients should not have a strong expectation that one or other method should lead to a more favourable outcome. The success of blinding will be assessed using the Bang Blinding Index (BBI) (24).

Outcomes

The primary outcome is length of hospital stay, from start of surgery to hospital discharge. The secondary outcomes include:

1. Acute post-operative pain assessed using the numerical rating scale (NRS) completed at 1 hr, 4 hr, 12 hr post-surgery and then twice daily until discharge;
2. Opioid consumption in the period from: i) surgery until hospital discharge; ii) discharge until 4 months;
3. Adverse health events in the period from: i) randomisation to discharge; ii) discharge until 4 months;

For peer review only

4. HRQoL measured using the EuroQol 5 dimension 5 level questionnaire (EQ-5D 5L) and Short-form (SF) 12 completed at baseline, 4 weeks and 4 months;
5. Resource use to 4 months (measured during the hospital stay, at 4 weeks and 4 months);
6. Chronic pain measured using the brief pain inventory (BPI) at baseline, at 4 weeks and 4 months.

Data collection

Screening data will be collected before consent to establish patient eligibility. The schedule of data collection outlined in Table 1 will take place after consent has been received. Data will be collected onto paper data collection forms, entered onto a bespoke study database and stored on a secure server. Patient reported questionnaire data is also stored on the study database. Data for the primary outcome and most secondary outcomes will be collected during the hospital stay. Patients will be followed up at approximately 4 weeks and at 4 months for information on pain, adverse events, resource use and quality of life.

The study will end for a participant after they have completed follow up at 4-months post-surgery. The end of the study as a whole will be after all study participants have completed follow up, all data queries have been resolved, the database locked, and the analysis completed.

For peer review only

Table 1. Schedule of data collection

Data item	Pre-randomisation	Pre-surgery	Intra-Operative	Post-surgery (until discharge)	Discharge	4 weeks post-surgery	4 months post-surgery
Socio-demo-graphic details	✓						
Co-morbidities	✓						
Routine clinical measures	✓				✓		
Resource use schedule					✓	✓	✓
SF-12	✓					✓	✓
EQ-5D 5L	✓					✓	✓
BPI	✓					✓	✓
NRS pain score	✓*			✓*	✓		
Study medication		✓		✓**			
Opioid use	✓		✓	✓	✓	✓	✓
Adverse events				✓	✓		
Serious adverse events				✓	✓	✓	✓

* Routinely collected NRS pain scores as close as possible to the following time points may be used: pre-randomisation, 1 hr, 4 hrs, 12 hrs post-surgery and twice daily post-surgery until discharge. NRS pain assessments will not be possible in intubated patients.

** Study medication given morning and evening for 2 days following extubation (where applicable).

For peer review only

Sample size

A total of 1500 participants will be randomised to either gabapentin or placebo. The target difference in length of hospital stay was chosen to reflect the effect size that would persuade clinicians to change practice and is expressed in terms of the increase in the proportion of patients discharged at the current median time to discharge (5 days for cardiac and abdominal surgery, 3 days for thoracic surgery). This sample size will have 90% power to detect a difference of 12.5% in each specialty (i.e. 50% versus 62.5%) if the number of participants per surgical stratum exceeds 376 and 80% power to detect a difference of 10% in each specialty (i.e. 50% versus 60%) if the number of participants per surgical stratum exceeds 430, assuming: 5% 2-sided type I error rate, 5% censoring, and constant hazard ratio.

Statistical analyses

The analyses will be conducted according to intention-to-treat and follow CONSORT reporting guidelines. Randomised participants who fail to complete the course of treatment will be included in the primary analysis. All models will compare the treatment groups, will be adjusted for centre and will include a treatment by speciality interaction so the treatment effect in each surgical specialty can be quantified and compared.

The primary outcome analysis of whether there is a difference between gabapentin and placebo with respect to length of hospital stay will use Cox proportional hazards regression. Those participants who die before discharge will be censored at the longest recorded length of stay for that specialty, as this is computationally equivalent to competing risk methodology in this setting.

For peer review only - http://bmjopen.bmj.com/site/about/guidelines.xhtml

Opioid consumption, pain scores and HRQoL outcomes will all be analysed using mixed regression models, adjusted for baseline measures where appropriate. Changes in treatment effect with time will be assessed by adding a treatment by time interaction to the model and comparing models using a likelihood ratio test. Deaths will be accounted for by modelling HRQoL and survival jointly. Model fit will be assessed and alternative models and/or transformations (e.g. to induce normality) will be explored where appropriate. Safety will be assessed by summarising the number and proportion of participants reporting serious and non-serious adverse events and will be reported to the Data Monitoring and Safety Committee (DMSC) on a regular basis.

The health economic evaluation will compare the costs and effects of gabapentin compared to placebo for the management of pain after major surgery. The within-trial cost-effectiveness analysis will be undertaken from an NHS and personal social services perspective, with a 4 month time horizon from the day of surgery. Effects will be measured using quality-adjusted life years (QALYs), estimated using EQ-5D 5L (25, 26). Costs will include medication costs and those related to inpatient stay. Established guidelines as set out by the National Institute for Health and Care Excellence (NICE) (27) will be followed for the economic evaluation. The incremental cost-effectiveness ratio will be calculated from the average costs and QALYs in each trial group to produce an incremental cost per QALY of gabapentin compared to placebo (28).

For peer review only - http://bmjopen.bmj.com/site/about/guidelines.xhtml

Exploratory subgroup analyses are planned to explore the primary and secondary outcomes in terms of type of surgery (open/minimal access).

Data handling, storage and sharing

Data will be stored in a bespoke database hosted on the NHS network. Access to the database will be via a secure password-protected web-interface. All study documentation will be retained in a secure location during the study and for 15 years after the end of the study, when all patient identifiable paper records will be destroyed by confidential means. Medical records documenting study related information will be identified by a label bearing the name and duration of the study. In compliance with the Medical Research Council Policy on Data Sharing, relevant 'meta'-data about the study and the full dataset, but without any participant identifiers other than the unique participant identifier, will be held indefinitely on a University of Bristol server. A secure electronic 'key' with a unique participant identifier, and key personal identifiers will also be held indefinitely, but in a separate file and in a physically different location (NHS hospital server). These will be retained because of the potential for the raw data to be used subsequently for secondary research.

For peer review only

Risk of bias

The following key features have been incorporated into the study to minimise the risk of bias:

1. Selection/allocation bias arising from the randomisation process will be prevented by using computer-generated concealed randomisation. Allocation lists prepared by an unblinded statistician will be stratified by centre and

For peer review only - http://bmjopen.bmj.com/site/about/guidelines.xhtml

specialty to minimise confounding. Participants will be randomised after eligibility is confirmed.

2. Performance bias arising from deviations from intended interventions will be minimised by blinding all participants, clinicians and other hospital staff caring for participants and members of the research team (apart from the study unblinded statistician) to participants' allocation. The success of blinding will be assessed by asking participants and research nurses responsible for participant care and data collection to complete the BBI at the point of discharge from hospital. Participants will complete the BBI again 4 months after surgery (24). Performance bias will also be minimised by administering the study medication according to standard protocols and by pre-defining all other study procedures and applying these to all participants in the same way. Adherence to all aspects of the protocol will be monitored.
3. Detection bias arising from differences in how the outcome is measured will be minimised by blinding all individuals assessing outcomes, assessing the success of blinding and providing clear unambiguous definitions for each outcome measure.
4. Attrition bias arising from missing outcome data will be minimised by i) maintaining contact with participants throughout the duration of the study to maximise the proportion of participants for whom all outcome data are available, ii) implementing measures to promote adherence (e.g. training for staff administering the intervention, posters to remind the care team of patient study participation) and iii) documenting non-adherence to the allocated treatment. The data will also be analysed by intention-to-treat. In estimating the target sample size, loss to follow-up has not been allowed for as the

For peer review only - http://bmjopen.bmj.com/site/about/guidelines.xhtml

primary outcome is time to hospital discharge and the follow-up period is short (4 months). However, attention will be paid to keeping in touch with participants and maximising retention up to 4 months.

5. Reporting bias will be minimised by having pre-specified outcomes and a pre-specified analysis plan.

Patient and Public Involvement (PPI)

PPI input was sought at the study design phase from relevant surgical PPI groups: the NIHR Bristol Biomedical Research Centre (Nutrition) colorectal PPI group, the Royal Brompton Hospital Cancer Consortia PPI group and patients who underwent cardiac surgery at the Bristol Heart Institute. GAP also includes a patient co-applicant. All PPI groups and the patient co-applicant unanimously agreed that the study was important and welcomed treatments that might reduce the amount of opioid drugs patients receive, and their associated side effects, after surgery. PPI groups provided feedback on the study intervention and outcome data collection (e.g. pain scores and questionnaires), which informed the study design.

PPI engagement will continue during study implementation, including writing and designing participant-facing documents and outlining the participant follow up schedule. The GAP study Trial Steering Committee (TSC) includes two public members who regularly review study progress.

PPI groups will continue to help with all aspects of the study, including preparing lay results summaries for dissemination to participants and other patient groups in order to maximise public awareness of the findings.

For peer review only - http://bmjopen.bmj.com/site/about/guidelines.xhtml

ETHICS AND DISSEMINATION

The study received Research Ethics Committee (REC) approval from Yorkshire and the Humber - Sheffield REC in November 2017, Medicines and Healthcare Regulatory Agency approval in December 2017 and Health Research Authority (HRA) approval in January 2018.

The study is sponsored by University Hospitals Bristol and Weston NHS Foundation Trust (www.uhbristol.nhs.uk/research-innovation/) and is coordinated by the Bristol Trials Centre, Clinical Trials and Evaluation Unit, (BTC (CTEU)), a UK Clinical Research Collaboration registered Clinical Trials Unit (reference 11). The TSC is made up of representatives from the GAP study team and independent members approved by the funder. The DMSC consists of an independent medical statistician and medical experts in this field approved by the funder. The TSC and DMSC meet as frequently as they feel is necessary, usually at least once a year.

Changes to the protocol since REC/HRA approval

Following REC and HRA approval several changes have been made to the study protocol, as follows: i) safety reporting requirement updates; ii) reference safety drug information updates; iii) clarifications about the level of care provided to study participants; iv) clarifications that patients must be expected to be able to swallow during the time of the study intervention to be eligible; v) clarification that the first post-operative dose should only be administered if patients are clinically able to swallow; vi) study medication packs contain 6 capsules instead of 8 (to minimise the chance of participants receiving more study medication doses than intended); vii)

For peer review only

permitting eligibility and prescription sign off by non-doctor clinicians (e.g. nurse practitioners); viii) provision of optional patient diaries; ix) opening to recruitment from more centres; and x) study team contact detail updates. Protocol version 8.0 (dated 03/12/2019) is currently in use.

Dissemination of findings

Findings will be disseminated to participating hospitals and to the academic community through peer reviewed publications and presentation at national and international meetings. Findings will also be shared with study participants who express a wish to receive study results through patient organisations, leaflets and newsletters. Study updates are regularly provided to the study team, participants and members of the public though emails, newsletters, magazine articles and social media.

DISCUSSION

The study design, involving three surgical specialties, was chosen because it is efficient and maximises the value of the research for the NHS. The inclusion of different surgical specialties reflects current clinical practice (gabapentin is prescribed to patients undergoing different surgical procedures) and should make the trial results generalisable in the NHS. The study opened to recruitment on 12/04/2018 and is currently recruiting in six centres. To date, 853 patients have been recruited (469 cardiac, 221 thoracic, 163 abdominal). The progression criteria were met and approvals to progress to phase 2 were received on 04/03/2019. GAP has proven more difficult to deliver than anticipated for a study which was perceived to have a straightforward intervention. Patient eligibility and patient willingness to

For peer review only - http://bmjopen.bmj.com/site/about/guidelines.xhtml

participate have not been a limit to recruitment. Some of the challenges of delivering the study include i) higher than expected training requirements to integrate administration of study medication into routine clinical practice in all specialties at participating centres, ii) difficulties of using multiple clinical prescribing systems (electronic and paper) which are not linked and require multiple manual updates for a single in-hospital patient stay, iii) higher than anticipated research team resource required to meet regulatory requirements (e.g. obtaining clinician eligibility sign off, often during unsocial hours, or additional requirements following the reclassification of gabapentin as a schedule 3 controlled drug in April 2019); and iv) regulatory structures that do not permit the study to have a designated principal investigator for each speciality in a centre. This is particularly challenging when patients are under the care of different clinical teams that are administratively and geographically separate. Further details about the challenges of delivering the GAP study in an NHS setting will be reported elsewhere. This study highlights that while the design is methodologically attractive, the current regulatory structures and NHS systems make implementation sub-optimal.

For peer review only

AUTHOR CONTRIBUTIONS

SB is involved in conducting the trial and assembled the manuscript from the trial protocol. MP identified the funding opportunity and designed the trial with statistical input from CR. CR is the non-clinical lead and the methodology/statistics lead for the trial. BG is the chief investigator and NC is the clinical pain lead for the trial. LCo drafted the statistical analysis plan. SW is the health economics lead and ES the health economist working on the trial. SB and ES designed the data collection for the health economics element of the trial. LC provides senior trial management

For peer review only - http://bmjopen.bmj.com/site/about/guidelines.xhtml

oversight and advice. HM, SDJ and JL have assisted with the set-up and delivery of the trial. MAH, RA, AA, GC, ME, NG and MM are participating clinicians in the trial. All authors have been involved in preparation of the study protocol and have read and approved the final manuscript.

ACKNOWLEDGEMENTS

The GAP study is sponsored by University Hospitals Bristol and Weston NHS Foundation Trust. This study was designed and delivered in collaboration with the BTC (CTEU), a UKCRC registered clinical trials unit which is in receipt of National Institute for Health Research Clinical Trial Unit support funding. The authors would like to thank all the research and clinical team members involved in recruitment, coordination, delivery of the intervention, data collection and data entry for this study.

COMPETING INTERESTS STATEMENT

No competing interests are declared. At the time that the work was carried out, GC was employed by the University Hospitals Bristol NHS Foundation Trust, Bristol, UK. GC is currently the Medical Director Johnson and Johnson Medical Devices UK and Ireland. GC has no competing interests to declare.

FUNDING STATEMENT

This study is funded by the National Institute for Health Research Health Technology Assessment Programme (15/101/16). The views and opinions expressed therein are those of the author(s) and do not necessarily reflect those of the National Institute of Health Research, NHS, or the Department of Health and Social Care.

For peer review only - http://bmjopen.bmj.com/site/about/guidelines.xhtml

LIST OF ABBREVIATIONS

BBI	Bang Blinding Index
BPI	Brief pain inventory
CTEU	Clinical Trials and Evaluation Unit
DMSC	Data monitoring and safety committee
eGFR	Estimated glomerular filtration rate: derived from gender, age, ethnicity and serum creatinine
EQ-5D 5L	EuroQol 5 dimension 5 level questionnaire
HRA	Health Research Authority
HRQoL	Health-related quality of life
GCP	Good clinical practice
MHRA	Medicines and healthcare products regulatory agency
NRS	Numerical rating score
NSAIDs	Non-steroidal anti-inflammatory drugs
PIL	Patient information leaflet
QALYs	Quality-adjusted life years
RCT	Randomised controlled trial
REC	Research ethics committee
SF-12	Short-form-12
TSC	Trial steering committee

Figure 1. Trial Schema

For peer review only

REFERENCES

1. Dumville JC, Gray TA, Walter CJ, Sharp CA, Page T, Macefield R, et al. Dressings for the prevention of surgical site infection. The Cochrane database of systematic reviews. 2016;12:Cd003091.
2. Apfelbaum JL, Chen C, Mehta SS, Gan TJ. Postoperative pain experience: results from a national survey suggest postoperative pain continues to be undermanaged. Anesth Analg. 2003;97(2):534-40.
3. Gan TJ, Habib AS, Miller TE, White W, Apfelbaum JL. Incidence, patient satisfaction, and perceptions of post-surgical pain: results from a US national survey. Current medical research and opinion. 2014;30(1):149-60.
4. Doleman B, Heinink TP, Read DJ, Faleiro RJ, Lund JN, Williams JP. A systematic review and meta-regression analysis of prophylactic gabapentin for postoperative pain. Anaesthesia. 2015;70(10):1186-204.
5. Chaparro LE, Smith SA, Moore RA, Wiffen PJ, Gilron I. Pharmacotherapy for the prevention of chronic pain after surgery in adults. The Cochrane database of systematic reviews. 2013;7:Cd008307.
6. Dolin SJ, Cashman JN, Bland JM. Effectiveness of acute postoperative pain management: I. Evidence from published data. Br J Anaesth. 2002;89(3):409-23.
7. Katz J, Seltzer Z. Transition from acute to chronic postsurgical pain: risk factors and protective factors. Expert Rev Neurother. 2009;9(5):723-44.
8. Chapman CR, Vierck CJ. The Transition of Acute Postoperative Pain to Chronic Pain: An Integrative Overview of Research on Mechanisms. The journal of pain : official journal of the American Pain Society. 2017;18(4):359.e1-.e38.
9. Kinney MA, Hooten WM, Cassivi SD, Allen MS, Passe MA, Hanson AC, et al. Chronic postthoracotomy pain and health-related quality of life. Ann Thorac Surg. 2012;93(4):1242-7.
10. Alam A, Gomes T, Zheng H, Mamdani MM, Juurlink DN, Bell CM. Long-term analgesic use after low-risk surgery: a retrospective cohort study. Arch Intern Med. 2012;172(5):425-30.
11. Clarke H, Soneji N, Ko DT, Yun L, Wijeysundera DN. Rates and risk factors for prolonged opioid use after major surgery: population based cohort study. BMJ (Clinical research ed). 2014;348:g1251.
12. https://www.surveymonkey.co.uk/r/Gabapentinoids_survey, accessed 04/05/2020. Gabapentinoids for acute operative pain_final survey
13. Nicholson A, Lowe MC, Parker J, Lewis SR, Alderson P, Smith AF. Systematic review and meta-analysis of enhanced recovery programmes in surgical patients. Br J Surg. 2014;101(3):172-88.
14. Straube S, Derry S, Moore RA, Wiffen PJ, McQuay HJ. Single dose oral gabapentin for established acute postoperative pain in adults. The Cochrane database of systematic reviews. 2010(5):Cd008183.
15. Alayed N, Alghanaim N, Tan X, Tulandi T. Preemptive use of gabapentin in abdominal hysterectomy: a systematic review and meta-analysis. Obstet Gynecol. 2014;123(6):1221-9.
16. Yu L, Ran B, Li M, Shi Z. Gabapentin and pregabalin in the management of postoperative pain after lumbar spinal surgery: a systematic review and meta-analysis. Spine (Phila Pa 1976). 2013;38(22):1947-52.
17. Han C, Li XD, Jiang HQ, Ma JX, Ma XL. The use of gabapentin in the management of postoperative pain after total knee arthroplasty: A PRISMA-compliant meta-analysis of randomized controlled trials. Medicine. 2016;95(23):e3883.
18. Hwang SH, Park IJ, Cho YJ, Jeong YM, Kang JM. The efficacy of gabapentin/pregabalin in improving pain after tonsillectomy: A meta-analysis. The Laryngoscope 2016;126(2):357-66.

For peer review only - http://bmjopen.bmj.com/site/about/guidelines.xhtml

19. Maitra S, Baidya DK, Bhattacharjee S, Som A. Perioperative gabapentin and pregabalin in cardiac surgery: a systematic review and meta-analysis. Revista Brasileira de Anestesiologia. 2017;67(3):294-304.
20. Sanders JG, Dawes PJ. Gabapentin for Perioperative Analgesia in OtorhinolaryngologyHead and Neck Surgery: Systematic Review. Otolaryngology-head and neck surgery : official journal of American Academy of Otolaryngology-Head and Neck Surgery. 2016;155(6):893-903.
21. Zhai L, Song Z, Liu K. The Effect of Gabapentin on Acute Postoperative Pain in Patients Undergoing Total Knee Arthroplasty: A Meta-Analysis. Medicine. 2016;95(20):e3673.
22. Felder L, Saccone G, Scuotto S, Monks DT, Carvalho JCA, Zullo F, et al. Perioperative gabapentin and post cesarean pain control: A systematic review and meta-analysis of randomized controlled trials. European Journal of Obstetrics, Gynecology and Reproductive Biology. 2019 233:98-106.
23. Toma O, Persoons B, Pogatzki-Zahn E, Van de Velde M, Joshi GP, collaborators PWG. PROSPECT guideline for rotator cuff repair surgery: systematic review and procedure-specific postoperative pain management recommendations. Anaesthesia. 2019;74(10):1320-31.
24. Bang H, Ni L, Davis CE. Assessment of blinding in clinical trials. Controlled Clinical Trials 25. 2004:143-56.
25. Dolan P. Modeling valuations for EuroQol health states. Medical care. 1997 35(11):1095-108.
26. Herdman M, Gudex C, Lloyd A, Janssen MF, Kind P, Parkin D, et al. Development and preliminary testing of the new five-level version of EQ-5D (EQ-5D-5L). Qual Life Res. 2011;20(10):1727-36.
27. National Institute for Health and Care Excellence. Guide to the methods of technology appraisal 2013. Available from: http://publications.nice.org.uk/pmg9.
28. Gray A CP, Wolstenholme J, Wordsworth S. Applied Methods of Cost-Effectiveness Analysis in Health Care. In: Gray A, Briggs S, editors. Handbooks in Health Economic Evaluation Series. Oxford: Oxford University Press; 2011.

For peer review only

For peer review only - http://bmjopen.bmj.com/site/about/guidelines.xhtml

Figure 1. Trial Schema

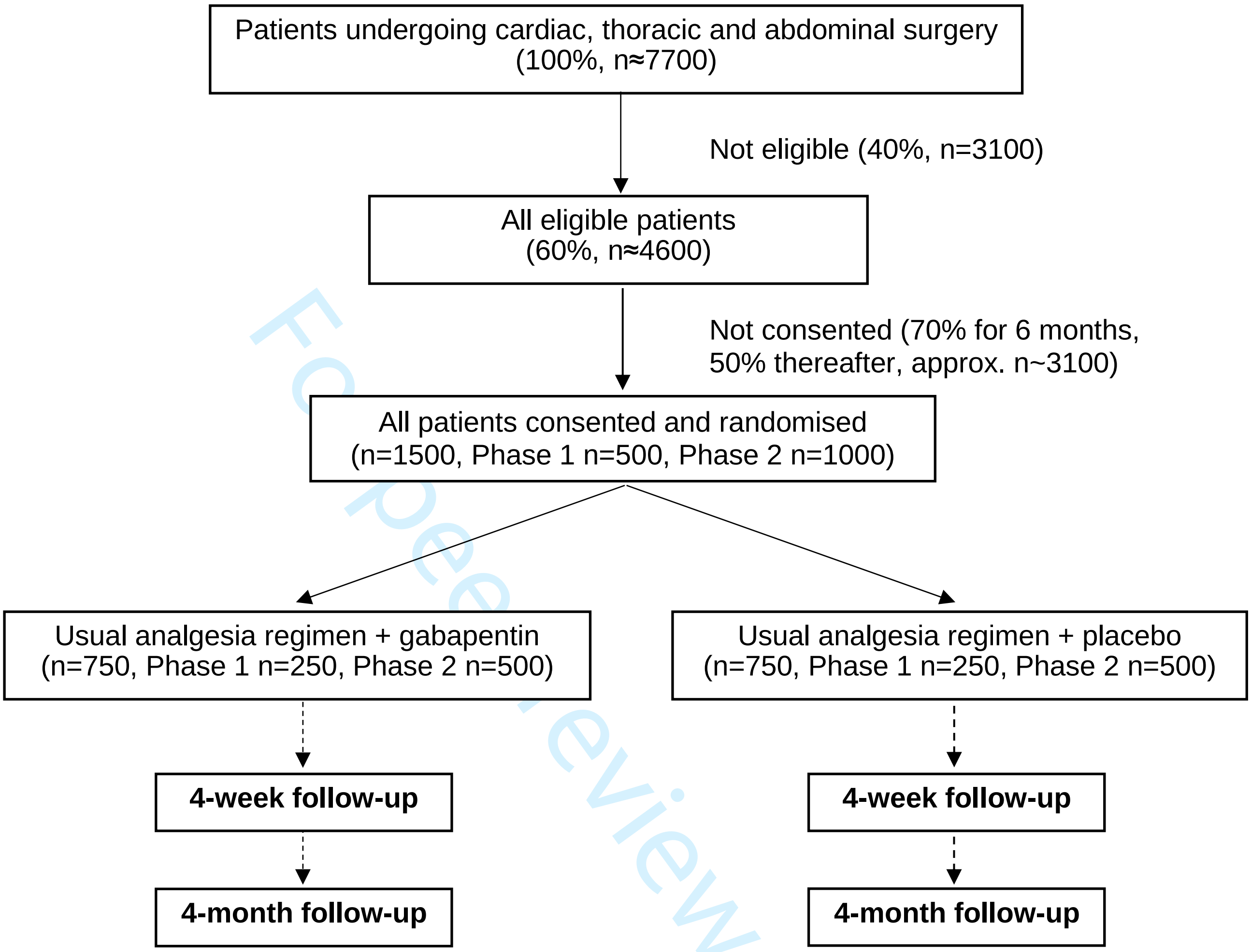

For peer review only

For peer review only - http://bmjopen.bmj.com/site/about/guidelines.xhtml

STANDARD PROTOCOL ITEMS: RECOMMENDATIONS FOR INTERVENTIONAL TRIALS

SPIRIT 2013 Checklist: Recommended items to address in a clinical trial protocol and related documents*

Section/item	Item No	Description	Addressed on page number
**Administrative information**			
Title	1	Descriptive title identifying the study design, population, interventions, and, if applicable, trial acronym	1
Trial registration	2a	Trial identifier and registry name. If not yet registered, name of intended registry	3
	2b	All items from the World Health Organization Trial Registration Data Set	N/A
Protocol version	3	Date and version identifier	20
Funding	4	Sources and types of financial, material, and other support	22
21Roles and responsibilities	5a	Names, affiliations, and roles of protocol contributors	1-2, 21-22
	5b	Name and contact information for the trial sponsor	19
	5c	Role of study sponsor and funders, if any, in study design; collection, management, analysis, and interpretation of data; writing of the report; and the decision to submit the report for publication, including whether they will have ultimate authority over any of these activities	22
	5d	Composition, roles, and responsibilities of the coordinating centre, steering committee, endpoint adjudication committee, data management team, and other individuals or groups overseeing the trial, if applicable (see Item 21a for data monitoring committee)	19, 22

For peer review only

**Introduction**			
Background and rationale	6a	Description of research question and justification for undertaking the trial, including summary of relevant studies (published and unpublished) examining benefits and harms for each intervention	4-5
	6b	Explanation for choice of comparators	4-5
Objectives	7	Specific objectives or hypotheses	5-6
Trial design	8	Description of trial design including type of trial (eg, parallel group, crossover, factorial, single group), allocation ratio, and framework (eg, superiority, equivalence, noninferiority, exploratory)	6
**Methods: Participants, interventions, and outcomes**			
Study setting	9	Description of study settings (eg, community clinic, academic hospital) and list of countries where data will be collected. Reference to where list of study sites can be obtained	6. List of current sites upon request gap-study@bristol.ac.uk
Eligibility criteria	10	Inclusion and exclusion criteria for participants. If applicable, eligibility criteria for study centres and individuals who will perform the interventions (eg, surgeons, psychotherapists)	7-8
Interventions	11a	Interventions for each group with sufficient detail to allow replication, including how and when they will be administered	9
	11b	Criteria for discontinuing or modifying allocated interventions for a given trial participant (eg, drug dose change in response to harms, participant request, or improving/worsening disease)	8-9
	11c	Strategies to improve adherence to intervention protocols, and any procedures for monitoring adherence (eg, drug tablet return, laboratory tests)	6,9
	11d	Relevant concomitant care and interventions that are permitted or prohibited during the trial	9

For peer review only - http://bmjopen.bmj.com/site/about/guidelines.xhtml

Outcomes	12	Primary, secondary, and other outcomes, including the specific measurement variable (eg, systolic blood pressure), analysis metric (eg, change from baseline, final value, time to event), method of aggregation (eg, median, proportion), and time point for each outcome. Explanation of the clinical relevance of chosen efficacy and harm outcomes is strongly recommended	11,12
Participant timeline	13	Time schedule of enrolment, interventions (including any run-ins and washouts), assessments, and visits for participants. A schematic diagram is highly recommended	8-10,12, Table 1 and Figure 1
Sample size	14	Estimated number of participants needed to achieve study objectives and how it was determined, including clinical and statistical assumptions supporting any sample size calculations	13
Recruitment	15	Strategies for achieving adequate participant enrolment to reach target sample size	6,18,20-21
**Methods: Assignment of interventions (for controlled trials)**			
Allocation:			
Sequence generation	16a	Method of generating the allocation sequence (eg, computer-generated random numbers), and list of any factors for stratification. To reduce predictability of a random sequence, details of any planned restriction (eg, blocking) should be provided in a separate document that is unavailable to those who enrol participants or assign interventions	10
Allocation concealment mechanism	16b	Mechanism of implementing the allocation sequence (eg, central telephone; sequentially numbered, opaque, sealed envelopes), describing any steps to conceal the sequence until interventions are assigned	10
Implementation	16c	Who will generate the allocation sequence, who will enrol participants, and who will assign participants to interventions	10
Blinding (masking)	17a	Who will be blinded after assignment to interventions (eg, trial participants, care providers, outcome assessors, data analysts), and how	10
	17b	If blinded, circumstances under which unblinding is permissible, and procedure for revealing a participant's allocated intervention during the trial	10
**Methods: Data collection, management, and analysis**			

Data collection methods	18a	Plans for assessment and collection of outcome, baseline, and other trial data, including any related processes to promote data quality (eg, duplicate measurements, training of assessors) and a description of study instruments (eg, questionnaires, laboratory tests) along with their reliability and validity, if known. Reference to where data collection forms can be found, if not in the protocol	12, Table 1
	18b	Plans to promote participant retention and complete follow-up, including list of any outcome data to be collected for participants who discontinue or deviate from intervention protocols	8,11-12,16-17
Data management	19	Plans for data entry, coding, security, and storage, including any related processes to promote data quality (eg, double data entry; range checks for data values). Reference to where details of data management procedures can be found, if not in the protocol	12,15-16
Statistical methods	20a	Statistical methods for analysing primary and secondary outcomes. Reference to where other details of the statistical analysis plan can be found, if not in the protocol	14-15
	20b	Methods for any additional analyses (eg, subgroup and adjusted analyses)	15
	20c	Definition of analysis population relating to protocol non-adherence (eg, as randomised analysis), and any statistical methods to handle missing data (eg, multiple imputation)	14-15
**Methods: Monitoring**			
Data monitoring	21a	Composition of data monitoring committee (DMC); summary of its role and reporting structure; statement of whether it is independent from the sponsor and competing interests; and reference to where further details about its charter can be found, if not in the protocol. Alternatively, an explanation of why a DMC is not needed	19
	21b	Description of any interim analyses and stopping guidelines, including who will have access to these interim results and make the final decision to terminate the trial	6,20
Harms	22	Plans for collecting, assessing, reporting, and managing solicited and spontaneously reported adverse events and other unintended effects of trial interventions or trial conduct	12
Auditing	23	Frequency and procedures for auditing trial conduct, if any, and whether the process will be independent from investigators and the sponsor	19
**Ethics and dissemination**			

Research ethics approval	24	Plans for seeking research ethics committee/institutional review board (REC/IRB) approval	18
Protocol amendments	25	Plans for communicating important protocol modifications (eg, changes to eligibility criteria, outcomes, analyses) to relevant parties (eg, investigators, REC/IRBs, trial participants, trial registries, journals, regulators)	19
Consent or assent	26a	Who will obtain informed consent or assent from potential trial participants or authorised surrogates, and how (see Item 32)	8
	26b	Additional consent provisions for collection and use of participant data and biological specimens in ancillary studies, if applicable	8
Confidentiality	27	How personal information about potential and enrolled participants will be collected, shared, and maintained in order to protect confidentiality before, during, and after the trial	8,12,15,16
Declaration of interests	28	Financial and other competing interests for principal investigators for the overall trial and each study site	22
Access to data	29	Statement of who will have access to the final trial dataset, and disclosure of contractual agreements that limit such access for investigators	16
Ancillary and post-trial care	30	Provisions, if any, for ancillary and post-trial care, and for compensation to those who suffer harm from trial participation	N/A
Dissemination policy	31a	Plans for investigators and sponsor to communicate trial results to participants, healthcare professionals, the public, and other relevant groups (eg, via publication, reporting in results databases, or other data sharing arrangements), including any publication restrictions	20
	31b	Authorship eligibility guidelines and any intended use of professional writers	N/A
	31c	Plans, if any, for granting public access to the full protocol, participant-level dataset, and statistical code	16
**Appendices**			
Informed consent materials	32	Model consent form and other related documentation given to participants and authorised surrogates	Upon request gap-study@bristol.ac.uk

For peer review only - http://bmjopen.bmj.com/site/about/guidelines.xhtml

Biological specimens	33	Plans for collection, laboratory evaluation, and storage of biological specimens for genetic or molecular analysis in the current trial and for future use in ancillary studies, if applicable	N/A

*It is strongly recommended that this checklist be read in conjunction with the SPIRIT 2013 Explanation & Elaboration for important clarification on the items. Amendments to the protocol should be tracked and dated. The SPIRIT checklist is copyrighted by the SPIRIT Group under the Creative Commons "Attribution-NonCommercial-NoDerivs 3.0 Unported" license.

For peer review only

BMJ Open

Effectiveness, cost effectiveness and safety of gabapentin versus placebo as an adjunct to multimodal pain regimens in surgical patients: Protocol of a placebo controlled randomised controlled trial with blinding (GAP study)

Journal:	*BMJ Open*
Manuscript ID	bmjopen-2020-041176.R1
Article Type:	Protocol
Date Submitted by the Author:	14-Oct-2020

Complete List of Authors:	Baos, Sarah; University of Bristol, Bristol Trials Centre, Clinical Trials and Evaluation Unit<br>Rogers, Chris; University of Bristol, Bristol Trials Centre, Clinical Trials and Evaluation Unit<br>Abbadi, Reyad; University Hospitals Bristol and Weston NHS Foundation Trust<br>Alzetani, Aiman; University Hospital Southampton NHS Foundation Trust<br>Casali, Gianluca; University Hospitals Bristol and Weston NHS Foundation Trust<br>Chauhan, Nilesh; University of Bristol and Weston NHS Foundation Trust<br>Collett, Laura; University of Bristol, Bristol Trials Centre, Clinical Trials and Evaluation Unit<br>Culliford, Lucy; University of Bristol, Bristol Trials Centre, Clinical Trials and Evaluation Unit<br>de Jesus, Samantha; University of Bristol, Bristol Trials Centre, Clinical Trials and Evaluation Unit<br>Edwards, Mark; University Hospital Southampton NHS Foundation Trust, Department of Anaesthesia; NIHR Biomedical Research Centre, University Southampton NHS Foundation Trust and University of Southampton, Acute, Critical & Perioperative Care Research Group<br>Goddard, Nicholas; University Hospital Southampton NHS Foundation Trust<br>Lamb, Jennifer; University of Bristol, Bristol Trials Centre, Clinical Trials and Evaluation Unit<br>McKeon, Holly; University of Bristol, Bristol Trials Centre, Clinical Trials and Evaluation Unit<br>Molyneux, Mat; University of Bristol and Weston NHS Foundation Trust<br>Stokes, Elizabeth; University of Oxford, Health Economics Research Centre Nuffield Department of Population Health<br>Wordsworth, Sarah; University of Oxford, University of Oxford<br>Gibbison, Ben; University of Bristol and Weston NHS Foundation Trust; University of Bristol<br>Pufulete, Maria; University of Bristol, Bristol Trials Centre, Clinical Trials and Evaluation Unit

**Primary Subject Heading**:	Anaesthesia

Secondary Subject Heading:	Surgery
Keywords:	Clinical trials < THERAPEUTICS, Pain management < ANAESTHETICS, SURGERY

SCHOLARONE™ Manuscripts

I, the Submitting Author has the right to grant and does grant on behalf of all authors of the Work (as defined in the below author licence), an exclusive licence and/or a non-exclusive licence for contributions from authors who are: i) UK Crown employees; ii) where BMJ has agreed a CC-BY licence shall apply, and/or iii) in accordance with the terms applicable for US Federal Government officers or employees acting as part of their official duties; on a worldwide, perpetual, irrevocable, royalty-free basis to BMJ Publishing Group Ltd ("BMJ") its licensees and where the relevant Journal is co-owned by BMJ to the co-owners of the Journal, to publish the Work in this journal and any other BMJ products and to exploit all rights, as set out in our licence.

The Submitting Author accepts and understands that any supply made under these terms is made by BMJ to the Submitting Author unless you are acting as an employee on behalf of your employer or a postgraduate student of an affiliated institution which is paying any applicable article publishing charge ("APC") for Open Access articles. Where the Submitting Author wishes to make the Work available on an Open Access basis (and intends to pay the relevant APC), the terms of reuse of such Open Access shall be governed by a Creative Commons licence – details of these licences and which Creative Commons licence will apply to this Work are set out in our licence referred to above.

Other than as permitted in any relevant BMJ Author's Self Archiving Policies, I confirm this Work has not been accepted for publication elsewhere, is not being considered for publication elsewhere and does not duplicate material already published. I confirm all authors consent to publication of this Work and authorise the granting of this licence.

For peer review only

Effectiveness, cost effectiveness and safety of gabapentin versus placebo as an adjunct to multimodal pain regimens in surgical patients: Protocol of a placebo controlled randomised controlled trial with blinding (GAP study)

Sarah Baos[1] sarah.baos@bristol.ac.uk
Chris A Rogers[1] chris.rogers@bristol.ac.uk
Reyad Al-Ghnaniem Abbadi[2] reyad.abbadi@UHBristol.nhs.uk
Aiman Alzetani[3] aiman.alzetani@uhs.nhs.uk
Gianluca Casali[2] gcasali@its.jnj.com
Nilesh Chauhan[2] nilesh.chauhan@uhbristol.nhs.uk
Laura Collett[1] laura.collett@bristol.ac.uk
Lucy Culliford[1] lucy.culliford@bristol.ac.uk
Samantha E de Jesus[1] samantha.dejesus@bristol.ac.uk
Mark Edwards[4, 5] mark.edwards2@uhs.nhs.uk
Nick Goddard[3] nicholas.goddard@uhs.nhs.uk
Jennifer Lamb[1] jenny.lamb@bristol.ac.uk
Holly McKeon[1] holly.mckeon@bristol.ac.uk
Mat Molyneux[2] mat.molyneux@uhbristol.nhs.uk
Elizabeth Stokes[6] elizabeth.stokes@dph.ox.ac.uk
Sarah Wordsworth[6] sarah.wordsworth@dph.ox.ac.uk
Ben Gibbison[2, 7] ben.gibbison@bristol.ac.uk
Maria Pufulete[1] maria.pufulete@bristol.ac.uk

For peer review only - http://bmjopen.bmj.com/site/about/guidelines.xhtml

1. Bristol Trials Centre, Clinical Trials and Evaluation Unit, University of Bristol, Bristol, UK

2. University Hospitals Bristol and Weston NHS Foundation Trust, Bristol, UK

3. University Hospital Southampton NHS Foundation Trust, Southampton, UK

4. Department of Anaesthesia, University Hospital Southampton NHS Foundation Trust, Southampton, UK

5. Acute, Critical & Perioperative Care Research Group, NIHR Biomedical Research Centre, University Hospital Southampton NHS Foundation Trust / University of Southampton, Southampton, UK

6. University of Oxford, Oxford, UK

7. University of Bristol, Bristol, UK

Corresponding author: Sarah Baos, Bristol Trials Centre, Clinical Trials and Evaluation Unit, University of Bristol, Bristol, UK. Email: sarah.baos@bristol.ac.uk. Tel: 0117 342 2297

Word count: 4057 (excluding title page, abstract, figures and tables, acknowledgements, contributions and references)

Number of figures: 1

Number of tables: 1

Keywords: Randomised controlled trial (RCT); gabapentin; placebo; pain management; peri-operative; surgical patients

For peer review only

For peer review only - http://bmjopen.bmj.com/site/about/guidelines.xhtml

ABSTRACT

Introduction: Gabapentin is an antiepileptic drug currently licensed to treat epilepsy and neuropathic pain but has been used off-label to treat acute post-operative pain. The GAP study will compare the effectiveness, cost-effectiveness and safety of gabapentin as an adjunct to standard multimodal analgesia versus placebo for the management of pain after major surgery.

Methods and analysis: The GAP study is a multi-centre, double-blind, randomised controlled trial in patients aged 18 years and over, undergoing different types of major surgery (cardiac, thoracic or abdominal). Patients will be randomised in a 1:1 ratio to receive either gabapentin (600mg just before surgery and 600mg/day for 2 days after surgery) or placebo in addition to usual pain management for each type of surgery. Patients will be followed up daily until hospital discharge and then at 4 weeks and 4 months after surgery. The primary outcome is length of hospital stay following surgery. Secondary outcomes include pain, total opioid use, adverse health events, health related quality of life and costs.

Ethics and dissemination: This study has been approved by the National Research Ethics Service. Findings will be shared with participating hospitals and disseminated to the academic community through peer reviewed publications and presentation at national and international meetings. Patients will be informed of the results through patient organisations and participant newsletters.

Trial registration: ISRCTN63614165. Registered on 05/06/2017.

For peer review only

ARTICLE SUMMARY

Strengths and limitations of this study

- Pragmatic design integrated into standard care pathways
- First trial to assess the impact of gabapentin on hospital stay and quality of life
- Implemented in three types of major surgery: cardiac, thoracic and abdominal
- Non-variable dose and limited duration of intervention may reduce applicability (e.g. frail / infirm patients and patients requiring analgesia for longer than 2 days)
- Only includes major body cavity surgery, which reduces applicability to major orthopaedic surgery

INTRODUCTION

About 4.7 million patients undergo surgery in the UK each year [1]. Many patients experience significant pain after surgery and about 10% experience severe pain [2-5]. Inadequate pain management increases the length of hospital stay [6] and contributes to the development of chronic or persistent post-surgical pain [7, 8], which impacts on quality of life [9]. Current multimodal analgesic regimens include paracetamol, non-steroidal anti-inflammatory drugs (NSAIDs), regional analgesia (focused delivery of a local anaesthetic to a specific part of the body) and opioids [10].

Opioids are key analgesic agents for managing moderate to severe pain. However, they have poor efficacy in movement-associated pain and have significant side-effects including confusion, nausea, vomiting, itching, constipation and respiratory depression. These side effects can increase the length of hospital stay, delay overall recovery and reduce quality of life [9]. Reliance on opioids after surgery also

For peer review only

For peer review only - http://bmjopen.bmj.com/site/about/guidelines.xhtml

increases the risk of long-term use and opioid dependence [11, 12]. Gabapentin is an anti-epileptic drug currently licensed to treat epileptic convulsions and neuropathic pain. It is also commonly used off-label in the peri-operative setting to reduce opioid use without compromising pain control. We conducted a survey of UK practice amongst consultant anaesthetists in the South West of England and members of the British Pain Society Acute Pain Special Interest Group. We found that 35/145 (23%) of anaesthetists prescribe gabapentin to their patients, with large variation in practice across the UK [13]. Reducing opioid use after surgery to return patients to full health as quickly as possible, is one of the central tenets of enhanced recovery in the NHS [14]. However, there is currently no robust evidence to recommend the inclusion of gabapentin in enhanced recovery protocols.

There are over 130 randomised controlled trials (RCTs) that have investigated gabapentin versus placebo in different surgical populations. Most of these trials are small (<200 patients, median 80) and highly heterogeneous, both statistically and clinically. These RCTs have been included in 18 systematic reviews that aimed to assess the effectiveness of gabapentin versus placebo in the peri-operative period; 11 of these in surgical populations [15-25]. All reviews reached the same conclusions; that gabapentin reduced opioid consumption and post-operative pain scores at 24 hours ($P<0.001$), but none has assessed the impact on quality of life. The most recent systematic review was published since this study started [25] and assessed the impact of gabapentin on length of hospital stay in 8 trials which provided very low to moderate quality evidence and found no statistically significant difference in the length of hospital stay between the gabapentin and control group.

For peer review only

The GAP study will compare the effectiveness, cost-effectiveness and safety of gabapentin versus placebo as an adjunct to standard multimodal analgesia for the management of pain after surgery. Specific objectives are to estimate: i) the difference between groups in length of hospital stay following surgery; ii) the difference between groups in total opioid use, pain, adverse events and health-related quality of life (HRQoL); and iii) the cost effectiveness of gabapentin compared to usual care.

METHODS AND ANALYSIS

Trial design and population

The GAP study is a multi-centre, parallel group, placebo-controlled, pragmatic double-blind RCT. Patients will be recruited from three surgical specialties (cardiac, thoracic and abdominal) across several secondary care NHS centres (Figure 1). A principal investigator will be appointed in each centre and clinical leads will be identified for each specialty within each centre.

GAP includes two phases: i) Phase 1 (12 months) involves study set-up and recruitment from two NHS secondary centres (University Hospitals Bristol and Weston NHS Foundation Trust and University Hospitals Southampton NHS Foundation Trust) with integrated monitoring of the recruitment process to maximise recruitment and adherence with the study medication; ii) Phase 2 (18 months) continued recruitment using the optimum methods established in phase 1, opening additional centres (if required). Progression from phase 1 to phase 2 is contingent on demonstrating that after 9 months of recruitment in phase 1, sufficient numbers of

For peer review only - http://bmjopen.bmj.com/site/about/guidelines.xhtml

patients referred for surgery are eligible for the trial and can be enrolled to complete the main trial. Specifically:

1. At least 60% of patients undergoing surgery are considered eligible;
2. At least 50% of eligible patients consent to randomisation by 6 months of recruitment at each centre.

Eligibility criteria

Patients will be eligible for the study if all the following apply:

1. Over 18 years of age;
2. Undergoing non-emergency surgery:

   i) Cardiac (surgery on the heart and great vessels performed via midline sternotomy);

   ii) Thoracic (open or minimal access surgery on the lungs and surrounding tissues);

   iii) Abdominal (open or minimal access surgery within the abdominal cavity);
3. Expected to stay in hospital at least until day 2 after surgery (day 0 is day of surgery);
4. Expected to be able to swallow during the time of the study intervention.

Patients will be excluded from the study if any of the following apply:

1. Taking anti-epileptic medication(s);
2. Gabapentin allergy;
3. Already taking gabapentin or gabapentanoids;
4. Galactose intolerance, Lapp lactase deficiency or glucose-galactose malabsorption;
5. Planned epidural analgesia;

For peer review only - http://bmjopen.bmj.com/site/about/guidelines.xhtml

6. Intended use of any gabapentanoids in the peri-operative analgesic protocol other than the study medication (this includes but is not restricted to: pregabalin, enacarbil gabapentin, 4-methylpregabalin and phenibut);
7. Known renal impairment (estimated glomerular filtration rate (eGFR) <30ml/min/1.73^2);
8. Weight <50kg;
9. Inability to provide written informed consent;
10. Unwilling to participate in follow-up;
11. Prisoners;
12. Enrolled in another clinical trial and: i) the patient is currently taking an investigational medicinal product as part of the other trial; or ii) co-enrolment is not permitted by the other trial; or iii) co-enrolment would be burdensome for the patient.

Patient approach and consent

Potential patients will be identified from clinic and planned operating lists and those eligible to participate will receive a patient information leaflet (PIL). Most patients will have at least 24 hours to consider participation. However, it is important to include urgent, non-emergency patients who may have less than 24 hours to consider the study, to maximise the applicability of the findings. In these circumstances, patients will only be enrolled if they confirm that they have had enough time to consider their participation.

Prior to surgery, patients will be seen by a member of the local research team who will answer any questions, confirm eligibility and receive written informed consent if

For peer review only - http://bmjopen.bmj.com/site/about/guidelines.xhtml

the patient decides to participate. Details of all patients approached and reasons for non-participation (e.g. ineligibility or patient refusal) will be documented. The patients' General Practitioners will be informed of their enrolment in the study. Participants can withdraw at any time and will be treated according to standard hospital procedures. If a participant decides that they no longer wish to take part in study procedures, data collection for those procedures will cease. These participants will be asked whether they are still willing to participate in the study follow-up, if applicable.

Interventions

The study intervention is gabapentin 600 mg given preoperatively and 600 mg/day (300 mg in the morning and 300 mg in the evening) given postoperatively for 2 days when clinically able to swallow following extubation (if applicable). The control is a placebo, taken at the same time-points as the active tablet. Both gabapentin and placebo will be administered within local multimodal analgesic regimens. The study medication (gabapentin/placebo) is manufactured, packaged and labelled in accordance with Good Manufacturing Practice and is stored at room temperature, below 25ºC.

Use of any gabapentanoids other than the study medication during the study intervention period is prohibited. If the pre-operative dose is administered and surgery is postponed by more than 12 hours, a second pre-operative dose of study medication will be given before the re-scheduled surgery. If a post-operative dose of study medication is missed by less than 6 hours, patients should be given the missed dose and continue to the next scheduled dose as per the protocol. If a dose

For peer review only - http://bmjopen.bmj.com/site/about/guidelines.xhtml

of study medication is missed by 6 hours or more, patients should continue to the next scheduled dose and should not be given the missed dose. For patients intubated for longer than 48 hours after the end of surgery, no post-operative study medication should be administered. All other aspects of patient's care will be performed according to local practice.

Randomisation

Randomisation will be performed after eligibility has been confirmed, using a secure internet-based randomisation system to ensure allocation concealment. Patients will be allocated in a 1:1 ratio to either gabapentin or placebo. A computer-generated allocation sequence will be prepared by an the unblinded study statistician. The random allocation will be blocked with blocks of varying size and stratified by centre and specialty, so that each specialty at each centre will have approximately equal numbers of patients allocated to placebo and gabapentin. To maintain blinding, the randomisation system will only reveal a unique pack number, which identifies the study medication to be given.

Blinding

Patients, their clinical care team (i.e. surgeon, anaesthetist, and those responsible for their post-operative care) and the research nurses will not be informed of the allocation. Patients will be made aware before entering the study that they will not be told which treatment they will receive. Doctors will prescribe 'study medication' rather than specifically gabapentin or placebo. The unique pack number provided by the randomisation system will provide the medication as specified by the pre-determined randomisation list. The allocations will only be known by pharmacy and

For peer review only - http://bmjopen.bmj.com/site/about/guidelines.xhtml

the unblinded study statistician and will not be disclosed to other members of the research team. The treatment allocation will only be unblinded if clinically indicated; for example, in the event of a suspected serious adverse reaction to the study medication, the management of which might be altered by knowledge of the allocation.

The study medication is over-encapsulated to maintain blinding. The capsules for active drug and placebo will look identical and do not have a particularly strong or unusual smell or taste, so we do not anticipate unblinding will occur due to the characteristics of the medication. Gabapentin may induce side-effects that may inadvertently unblind patients and/or clinical teams. However, given that the side effects of gabapentin (e.g. drowsiness, dizziness and difficulty concentrating) are similar to those of opioids, and that patients/clinical care teams are likely to view side effects as resulting from their whole surgical and post-operative experience, it is unlikely that any patient/clinician will definitively be able to attribute a specific side effect to gabapentin. The PIL and the process of informed consent explain the uncertainty around the potential beneficial effects of gabapentin over a placebo. Therefore, in the event of inadvertent unblinding, patients should not have a strong expectation that one or other method should lead to a more favourable outcome. The success of blinding will be assessed using the Bang Blinding Index (BBI) [26].

Outcomes

The primary outcome is length of hospital stay, from start of surgery to hospital discharge. The secondary outcomes include:

For peer review only - http://bmjopen.bmj.com/site/about/guidelines.xhtml

1. Acute post-operative pain assessed using the numerical rating scale (NRS) completed at rest and on movement (on mobilisation, deep breathing or coughing) at 1 hr, 4 hr, 12 hr post-surgery and then twice daily until discharge;
2. Opioid consumption in the period from: i) surgery until hospital discharge; ii) discharge until 4 months;
3. Adverse health events in the period from: i) randomisation to discharge; ii) discharge until 4 months;
4. HRQoL measured using the EuroQol 5 dimension 5 level questionnaire (EQ-5D 5L) and Short-form (SF) 12 completed at baseline, 4 weeks and 4 months;
5. Resource use to 4 months (measured during the hospital stay, at 4 weeks and 4 months);
6. Chronic pain measured using the brief pain inventory (BPI) at baseline, at 4 weeks and 4 months.

Data collection

Screening data will be collected before consent to establish patient eligibility. The schedule of data collection outlined in Table 1 will take place after consent has been received. Data will be collected onto paper data collection forms, entered onto a bespoke study database and stored on a secure server. Patient reported questionnaire data is also stored on the study database. Data for the primary outcome and most secondary outcomes will be collected during the hospital stay. Patients will be followed up at approximately 4 weeks and at 4 months for information on pain, adverse events, resource use and quality of life.

For peer review only

The study will end for a participant after they have completed follow up at 4-months post-surgery. The end of the study as a whole will be after all study participants have completed follow up, all data queries have been resolved, the database locked, and the analysis completed.

For peer review only

Table 1. Schedule of data collection

Data item	Pre-randomisation	Pre-surgery	Intra-Operative	Post-surgery (until discharge)	Discharge	4 weeks post-surgery	4 months post-surgery
Socio-demo-graphic details	✓						
Co-morbidities	✓						
Routine clinical measures	✓				✓		
Resource use schedule					✓	✓	✓
SF-12	✓					✓	✓
EQ-5D 5L	✓					✓	✓
BPI	✓					✓	✓
NRS pain score	✓*			✓*	✓		
Study medication		✓		✓**			
Opioid use	✓		✓	✓	✓	✓	✓
Adverse events				✓	✓		
Serious adverse events				✓	✓	✓	✓

* Routinely collected NRS pain scores as close as possible to the following time points may be used: pre-randomisation, 1 hr, 4 hrs, 12 hrs post-surgery and twice daily post-surgery until discharge. NRS pain assessments will not be possible in intubated patients.

** Study medication given morning and evening for 2 days following extubation (where applicable).

For peer review only

For peer review only - http://bmjopen.bmj.com/site/about/guidelines.xhtml

Sample size

A total of 1500 participants will be randomised to either gabapentin or placebo. The target difference in length of hospital stay was chosen to reflect the effect size that would persuade clinicians to change practice and is expressed in terms of the increase in the proportion of patients discharged at the current median time to discharge (5 days for cardiac and abdominal surgery, 3 days for thoracic surgery). This sample size will have 90% power to detect a difference of 12.5% in each specialty (i.e. 50% versus 62.5%) if the number of participants per surgical stratum exceeds 376 and 80% power to detect a difference of 10% in each specialty (i.e. 50% versus 60%) if the number of participants per surgical stratum exceeds 430, assuming: 5% 2-sided type I error rate, 5% censoring, and constant hazard ratio.

Statistical analyses

The analyses will be conducted according to intention-to-treat and follow CONSORT reporting guidelines. Randomised participants who fail to complete the course of treatment will be included in the primary analysis. All models will compare the treatment groups, will be adjusted for centre and will include a treatment by speciality interaction so the treatment effect in each surgical specialty can be quantified and compared.

The primary outcome analysis of whether there is a difference between gabapentin and placebo with respect to length of hospital stay will use Cox proportional hazards regression. Those participants who die before discharge will be censored at the longest recorded length of stay for that specialty, as this is computationally equivalent to competing risk methodology in this setting.

For peer review only

Opioid consumption, pain scores and HRQoL outcomes will all be analysed using mixed regression models, adjusted for baseline measures where appropriate. Changes in treatment effect with time will be assessed by adding a treatment by time interaction to the model and comparing models using a likelihood ratio test. Deaths will be accounted for by modelling HRQoL and survival jointly. Model fit will be assessed and alternative models and/or transformations (e.g. to induce normality) will be explored where appropriate. Safety will be assessed by summarising the number and proportion of participants reporting serious and non-serious adverse events and will be reported to the Data Monitoring and Safety Committee (DMSC) on a regular basis.

The health economic evaluation will compare the costs and effects of gabapentin compared to placebo for the management of pain after major surgery. The within-trial cost-effectiveness analysis will be undertaken from an NHS and personal social services perspective, with a 4 month time horizon from the day of surgery. Effects will be measured using quality-adjusted life years (QALYs), estimated using EQ-5D 5L [27, 28]. Costs will include medication costs and those related to inpatient stay. Established guidelines as set out by the National Institute for Health and Care Excellence (NICE) [29] will be followed for the economic evaluation. The incremental cost-effectiveness ratio will be calculated from the average costs and QALYs in each trial group to produce an incremental cost per QALY of gabapentin compared to placebo [30].

For peer review only

For peer review only - http://bmjopen.bmj.com/site/about/guidelines.xhtml

Exploratory subgroup analyses are planned to explore the primary and secondary outcomes in terms of type of surgery (open/minimal access).

Data handling, storage and sharing

Data will be stored in a bespoke database hosted on the NHS network. Access to the database will be via a secure password-protected web-interface. All study documentation will be retained in a secure location during the study and for 15 years after the end of the study, when all patient identifiable paper records will be destroyed by confidential means. Medical records documenting study related information will be identified by a label bearing the name and duration of the study. In compliance with the Medical Research Council Policy on Data Sharing, relevant 'meta'-data about the study and the full dataset, but without any participant identifiers other than the unique participant identifier, will be held indefinitely on a University of Bristol server. A secure electronic 'key' with a unique participant identifier, and key personal identifiers will also be held indefinitely, but in a separate file and in a physically different location (NHS hospital server). These will be retained because of the potential for the raw data to be used subsequently for secondary research.

For peer review only

Risk of bias

The following key features have been incorporated into the study to minimise the risk of bias:

1. Selection/allocation bias arising from the randomisation process will be prevented by using computer-generated concealed randomisation. Allocation lists prepared by an unblinded statistician will be stratified by centre and

For peer review only - http://bmjopen.bmj.com/site/about/guidelines.xhtml

specialty to minimise confounding. Participants will be randomised after eligibility is confirmed.

2. Performance bias arising from deviations from intended interventions will be minimised by blinding all participants, clinicians and other hospital staff caring for participants and members of the research team (apart from the study unblinded statistician) to participants' allocation. The success of blinding will be assessed by asking participants and research nurses responsible for participant care and data collection to complete the BBI at the point of discharge from hospital. Participants will complete the BBI again 4 months after surgery [26]. Performance bias will also be minimised by administering the study medication according to standard protocols and by pre-defining all other study procedures and applying these to all participants in the same way. Adherence to all aspects of the protocol will be monitored.
3. Detection bias arising from differences in how the outcome is measured will be minimised by blinding all individuals assessing outcomes, assessing the success of blinding and providing clear unambiguous definitions for each outcome measure.
4. Attrition bias arising from missing outcome data will be minimised by i) maintaining contact with participants throughout the duration of the study to maximise the proportion of participants for whom all outcome data are available, ii) implementing measures to promote adherence (e.g. training for staff administering the intervention, posters to remind the care team of patient study participation) and iii) documenting non-adherence to the allocated treatment. The data will also be analysed by intention-to-treat. In estimating the target sample size, loss to follow-up has not been allowed for as the

For peer review only - http://bmjopen.bmj.com/site/about/guidelines.xhtml

primary outcome is time to hospital discharge and the follow-up period is short (4 months). However, attention will be paid to keeping in touch with participants and maximising retention up to 4 months.

5. Reporting bias will be minimised by having pre-specified outcomes and a pre-specified analysis plan.

Patient and Public Involvement (PPI)

PPI input was sought at the study design phase from relevant surgical PPI groups: the NIHR Bristol Biomedical Research Centre (Nutrition) colorectal PPI group, the Royal Brompton Hospital Cancer Consortia PPI group and patients who underwent cardiac surgery at the Bristol Heart Institute. GAP also includes a patient co-applicant. All PPI groups and the patient co-applicant unanimously agreed that the study was important and welcomed treatments that might reduce the amount of opioid drugs patients receive, and their associated side effects, after surgery. PPI groups provided feedback on the study intervention and outcome data collection (e.g. pain scores and questionnaires), which informed the study design.

PPI engagement will continue during study implementation, including writing and designing participant-facing documents and outlining the participant follow up schedule. The GAP study Trial Steering Committee (TSC) includes two public members who regularly review study progress.

PPI groups will continue to help with all aspects of the study, including preparing lay results summaries for dissemination to participants and other patient groups in order to maximise public awareness of the findings.

For peer review only - http://bmjopen.bmj.com/site/about/guidelines.xhtml

ETHICS AND DISSEMINATION

The study received Research Ethics Committee (REC) approval from Yorkshire and the Humber - Sheffield REC in November 2017, Medicines and Healthcare Regulatory Agency approval in December 2017 and Health Research Authority (HRA) approval in January 2018.

The study is sponsored by University Hospitals Bristol and Weston NHS Foundation Trust (www.uhbristol.nhs.uk/research-innovation/) and is coordinated by the Bristol Trials Centre, Clinical Trials and Evaluation Unit, (BTC (CTEU)), a UK Clinical Research Collaboration registered Clinical Trials Unit (reference 11). The TSC is made up of representatives from the GAP study team and independent members approved by the funder. The DMSC consists of an independent medical statistician and medical experts in this field approved by the funder. The TSC and DMSC meet as frequently as they feel is necessary, usually at least once a year.

Changes to the protocol since REC/HRA approval

Following REC and HRA approval several changes have been made to the study protocol, as follows: i) safety reporting requirement updates; ii) reference safety drug information updates; iii) clarifications about the level of care provided to study participants; iv) clarifications that patients must be expected to be able to swallow during the time of the study intervention to be eligible; v) clarification that the first post-operative dose should only be administered if patients are clinically able to swallow; vi) study medication packs contain 6 capsules instead of 8 (to minimise the chance of participants receiving more study medication doses than intended); vii)

For peer review only - http://bmjopen.bmj.com/site/about/guidelines.xhtml

permitting eligibility and prescription sign off by non-doctor clinicians (e.g. nurse practitioners); viii) provision of optional patient diaries; ix) opening to recruitment from more centres; and x) study team contact detail updates. Protocol version 8.0 (dated 03/12/2019) is currently in use.

Dissemination of findings

Findings will be disseminated to participating hospitals and to the academic community through peer reviewed publications and presentation at national and international meetings. Findings will also be shared with study participants who express a wish to receive study results through patient organisations, leaflets and newsletters. Study updates are regularly provided to the study team, participants and members of the public though emails, newsletters, magazine articles and social media.

DISCUSSION

The study design, involving three surgical specialties, was chosen because it is efficient and maximises the value of the research for the NHS. The inclusion of different surgical specialties reflects current clinical practice (gabapentin is prescribed to patients undergoing different surgical procedures) and should make the trial results generalisable in the NHS. The study opened to recruitment on 12/04/2018 and is currently recruiting in six centres. To date, 853 patients have been recruited (469 cardiac, 221 thoracic, 163 abdominal). The progression criteria were met and approvals to progress to phase 2 were received on 04/03/2019. GAP has proven more difficult to deliver than anticipated for a study which was perceived to have a straightforward intervention. Patient eligibility and patient willingness to

For peer review only - http://bmjopen.bmj.com/site/about/guidelines.xhtml

participate have not been a limit to recruitment. Some of the challenges of delivering the study include i) higher than expected training requirements to integrate administration of study medication into routine clinical practice in all specialties at participating centres, ii) difficulties of using multiple clinical prescribing systems (electronic and paper) which are not linked and require multiple manual updates for a single in-hospital patient stay, iii) higher than anticipated research team resource required to meet regulatory requirements (e.g. obtaining clinician eligibility sign off, often during unsocial hours, or additional requirements following the reclassification of gabapentin as a schedule 3 controlled drug in April 2019); and iv) regulatory structures that do not permit the study to have a designated principal investigator for each speciality in a centre. This is particularly challenging when patients are under the care of different clinical teams that are administratively and geographically separate. Further details about the challenges of delivering the GAP study in an NHS setting will be reported elsewhere. This study highlights that while the design is methodologically attractive, the current regulatory structures and NHS systems make implementation sub-optimal.

AUTHOR CONTRIBUTIONS

SB is involved in conducting the trial and assembled the manuscript from the trial protocol. MP identified the funding opportunity and designed the trial with statistical input from CR. CR is the non-clinical lead and the methodology/statistics lead for the trial. BG is the chief investigator and NC is the clinical pain lead for the trial. LCo drafted the statistical analysis plan. SW is the health economics lead and ES the health economist working on the trial. SB and ES designed the data collection for the health economics element of the trial. LC provides senior trial management

For peer review only - http://bmjopen.bmj.com/site/about/guidelines.xhtml

oversight and advice. HM, SDJ and JL have assisted with the set-up and delivery of the trial. MAH, RA, AA, GC, ME, NG and MM are participating clinicians in the trial. All authors have been involved in preparation of the study protocol and have read and approved the final manuscript.

ACKNOWLEDGEMENTS

The GAP study is sponsored by University Hospitals Bristol and Weston NHS Foundation Trust. This study was designed and delivered in collaboration with the BTC (CTEU), a UKCRC registered clinical trials unit which is in receipt of National Institute for Health Research Clinical Trial Unit support funding. The authors would like to thank all the research and clinical team members involved in recruitment, coordination, delivery of the intervention, data collection and data entry for this study.

COMPETING INTERESTS STATEMENT

No competing interests are declared. At the time that the work was carried out, GC was employed by the University Hospitals Bristol NHS Foundation Trust, Bristol, UK. GC is currently the Medical Director Johnson and Johnson Medical Devices UK and Ireland. GC has no competing interests to declare.

FUNDING STATEMENT

This study is funded by the National Institute for Health Research Health Technology Assessment Programme (15/101/16). The views and opinions expressed therein are those of the author(s) and do not necessarily reflect those of the National Institute of Health Research, NHS, or the Department of Health and Social Care.

For peer review only - http://bmjopen.bmj.com/site/about/guidelines.xhtml

LIST OF ABBREVIATIONS

BBI	Bang Blinding Index
BPI	Brief pain inventory
CTEU	Clinical Trials and Evaluation Unit
DMSC	Data monitoring and safety committee
eGFR	Estimated glomerular filtration rate: derived from gender, age, ethnicity and serum creatinine
EQ-5D 5L	EuroQol 5 dimension 5 level questionnaire
HRA	Health Research Authority
HRQoL	Health-related quality of life
GCP	Good clinical practice
MHRA	Medicines and healthcare products regulatory agency
NRS	Numerical rating score
NSAIDs	Non-steroidal anti-inflammatory drugs
PIL	Patient information leaflet
QALYs	Quality-adjusted life years
RCT	Randomised controlled trial
REC	Research ethics committee
SF-12	Short-form-12
TSC	Trial steering committee

Figure 1. Trial Schema

For peer review only

REFERENCES

1. Dumville JC, Gray TA, Walter CJ, et al. Dressings for the prevention of surgical site infection. *Cochrane Database Syst Rev* 2016;12:Cd003091.
2. Apfelbaum JL, Chen C, Mehta SS, et al. Postoperative pain experience: results from a national survey suggest postoperative pain continues to be undermanaged. *Anesth Analg* 2003;97(2):534-40.
3. Gan TJ, Habib AS, Miller TE, et al, Incidence, patient satisfaction, and perceptions of post-surgical pain: results from a US national survey. *Curr Med Res Opin* 2014;30(1):149-60.
4. Doleman B, Heinink TP, Read DJ, et al. A systematic review and meta-regression analysis of prophylactic gabapentin for postoperative pain. *Anaesthesia* 2015;70(10):1186-204.
5. Chaparro LE, Smith SA, Moore RA, et al. Pharmacotherapy for the prevention of chronic pain after surgery in adults. *Cochrane Database Syst Rev* 2013;7:Cd008307.
6. Dolin SJ, Cashman JN, Bland JM. Effectiveness of acute postoperative pain management: I. Evidence from published data. *Br J Anaesth* 2002;89(3):409-23.
7. Katz J, Seltzer Z. Transition from acute to chronic postsurgical pain: risk factors and protective factors. *Expert Rev Neurother* 2009;9(5):723-44.
8. Chapman CR, Vierck CJ. The transition of acute postoperative pain to chronic pain: an integrative overview of research on mechanisms. *J Pain* 2017;18(4):359.e1-.e38.
9. Kinney MA, Hooten WM, Cassivi SD, et al. Chronic postthoracotomy pain and health-related quality of life. *Ann Thorac Surg* 2012;93(4):1242-7.
10. Feldheiser A, Aziz O, Baldini G, et al. Enhanced Recovery After Surgery (ERAS) for gastrointestinal surgery, part 2: consensus statement for anaesthesia practice. *Acta Anaesthesiol Scand* 2016;60(3):289-334.
11. Alam A, Gomes T, Zheng H, et al. Long-term analgesic use after low-risk surgery: a retrospective cohort study. *Arch Intern Med* 2012;172(5):425-30.
12. Clarke H, Soneji N, Ko DT, et al. Rates and risk factors for prolonged opioid use after major surgery: population based cohort study. *BMJ* 2014;348:g1251.
13. https://www.surveymonkey.co.uk/r/Gabapentinoids_survey, accessed October 2020. Gabapentinoids for acute operative pain_final survey
14. Nicholson A, Lowe MC, Parker J, et al. Systematic review and meta-analysis of enhanced recovery programmes in surgical patients. *Br J Surg* 2014;101(3):172-88.
15. Straube S, Derry S, Moore RA, et al. Single dose oral gabapentin for established acute postoperative pain in adults. *Cochrane Database Syst Rev* 2010(5):Cd008183.
16. Alayed N, Alghanaim N, Tan X, et al. Preemptive use of gabapentin in abdominal hysterectomy: a systematic review and meta-analysis. *Obstet Gynecol* 2014;123(6):1221-9.
17. Yu L, Ran B, Li M, et al. Gabapentin and pregabalin in the management of postoperative pain after lumbar spinal surgery: a systematic review and meta-analysis. *Spine (Phila Pa 1976)* 2013;38(22):1947-52.
18. Han C, Li XD, Jiang HQ, et al. The use of gabapentin in the management of postoperative pain after total knee arthroplasty: A PRISMA-compliant meta-analysis of randomized controlled trials. *Medicine* 2016;95(23):e3883.

For peer review only - http://bmjopen.bmj.com/site/about/guidelines.xhtml

19. Hwang SH, Park IJ, Cho YJ, et al. The efficacy of gabapentin/pregabalin in improving pain after tonsillectomy: A meta-analysis. *Laryngoscope* 2016;126(2):357-66.
20. Maitra S, Baidya DK, Bhattacharjee S, et al. Perioperative gabapentin and pregabalin in cardiac surgery: a systematic review and meta-analysis. *Rev Bras Anestesiol* 2017;67(3):294-304.
21. Sanders JG, Dawes PJ. Gabapentin for Perioperative Analgesia in Otorhinolaryngology Head and Neck Surgery: Systematic Review. *Otolaryngol Head Neck Surg* 2016;155(6):893-903.
22. Zhai L, Song Z, Liu K. The effect of gabapentin on acute postoperative pain in patients undergoing total knee arthroplasty: a meta-analysis. *Medicine* 2016;95(20):e3673.
23. Felder L, Saccone G, Scuotto S, et al. Perioperative gabapentin and post cesarean pain control: A systematic review and meta-analysis of randomized controlled trials. *Eur J Obstet Gynecol Reprod Biol* 2019 233:98-106.
24. Toma O, Persoons B, Pogatzki-Zahn E, et al. PROSPECT guideline for rotator cuff repair surgery: systematic review and procedure-specific postoperative pain management recommendations. *Anaesthesia* 2019;74(10):1320-31.
25. Verret M, Lauzier F, Zarychanski R, et al. Perioperative use of gabapentinoids for the management of postoperative acute pain a systematic review and meta-analysis. *Anesthesiology* 2020;133(2):265-79.
26. Bang H, Ni L, Davis CE. Assessment of blinding in clinical trials. *Control Clin Trials 25* 2004:143-56.
27. Dolan P. Modeling valuations for EuroQol health states. *Med Care* 1997 35(11):1095-108.
28. Herdman M, Gudex C, Lloyd A, et al. Development and preliminary testing of the new five-level version of EQ-5D (EQ-5D-5L). *Qual Life Res* 2011;20(10):1727-36.
29. National Institute for Health and Care Excellence. Guide to the methods of technology appraisal 2013. http://publications.nice.org.uk/pmg9, date accessed: October 2020.
30. Gray A CP, Wolstenholme J, Wordsworth S. Applied methods of cost-effectiveness analysis in health care. In: Gray A, Briggs S, editors. Handbooks in Health Economic Evaluation Series. Oxford: Oxford University Press; 2011.

For peer review only

For peer review only - http://bmjopen.bmj.com/site/about/guidelines.xhtml

Figure 1. Trial Schema

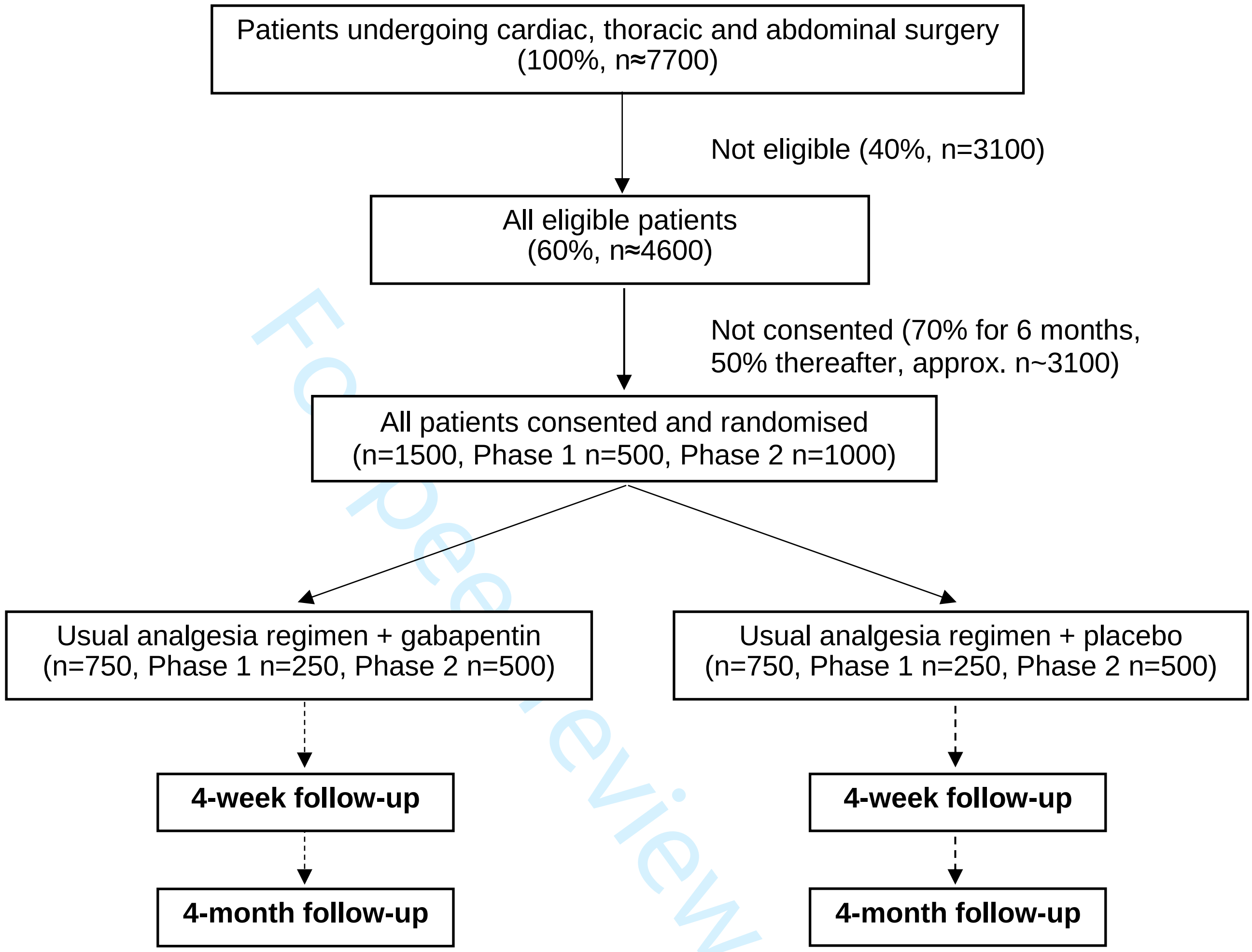

For peer review only - http://bmjopen.bmj.com/site/about/guidelines.xhtml

STANDARD PROTOCOL ITEMS: RECOMMENDATIONS FOR INTERVENTIONAL TRIALS

SPIRIT 2013 Checklist: Recommended items to address in a clinical trial protocol and related documents*

Section/item	Item No	Description	Addressed on page number
**Administrative information**			
Title	1	Descriptive title identifying the study design, population, interventions, and, if applicable, trial acronym	1
Trial registration	2a	Trial identifier and registry name. If not yet registered, name of intended registry	3
	2b	All items from the World Health Organization Trial Registration Data Set	N/A
Protocol version	3	Date and version identifier	20
Funding	4	Sources and types of financial, material, and other support	22
21Roles and responsibilities	5a	Names, affiliations, and roles of protocol contributors	1-2, 21-22
	5b	Name and contact information for the trial sponsor	19
	5c	Role of study sponsor and funders, if any, in study design; collection, management, analysis, and interpretation of data; writing of the report; and the decision to submit the report for publication, including whether they will have ultimate authority over any of these activities	22
	5d	Composition, roles, and responsibilities of the coordinating centre, steering committee, endpoint adjudication committee, data management team, and other individuals or groups overseeing the trial, if applicable (see Item 21a for data monitoring committee)	19, 22

For peer review only

**Introduction**			
Background and rationale	6a	Description of research question and justification for undertaking the trial, including summary of relevant studies (published and unpublished) examining benefits and harms for each intervention	4-5
	6b	Explanation for choice of comparators	4-5
Objectives	7	Specific objectives or hypotheses	5-6
Trial design	8	Description of trial design including type of trial (eg, parallel group, crossover, factorial, single group), allocation ratio, and framework (eg, superiority, equivalence, noninferiority, exploratory)	6
**Methods: Participants, interventions, and outcomes**			
Study setting	9	Description of study settings (eg, community clinic, academic hospital) and list of countries where data will be collected. Reference to where list of study sites can be obtained	6. List of current sites upon request gap-study@bristol.ac.uk
Eligibility criteria	10	Inclusion and exclusion criteria for participants. If applicable, eligibility criteria for study centres and individuals who will perform the interventions (eg, surgeons, psychotherapists)	7-8
Interventions	11a	Interventions for each group with sufficient detail to allow replication, including how and when they will be administered	9
	11b	Criteria for discontinuing or modifying allocated interventions for a given trial participant (eg, drug dose change in response to harms, participant request, or improving/worsening disease)	8-9
	11c	Strategies to improve adherence to intervention protocols, and any procedures for monitoring adherence (eg, drug tablet return, laboratory tests)	6,9
	11d	Relevant concomitant care and interventions that are permitted or prohibited during the trial	9

For peer review only - http://bmjopen.bmj.com/site/about/guidelines.xhtml

Outcomes	12	Primary, secondary, and other outcomes, including the specific measurement variable (eg, systolic blood pressure), analysis metric (eg, change from baseline, final value, time to event), method of aggregation (eg, median, proportion), and time point for each outcome. Explanation of the clinical relevance of chosen efficacy and harm outcomes is strongly recommended	11,12
Participant timeline	13	Time schedule of enrolment, interventions (including any run-ins and washouts), assessments, and visits for participants. A schematic diagram is highly recommended	8-10,12, Table 1 and Figure 1
Sample size	14	Estimated number of participants needed to achieve study objectives and how it was determined, including clinical and statistical assumptions supporting any sample size calculations	13
Recruitment	15	Strategies for achieving adequate participant enrolment to reach target sample size	6,18,20-21

Methods: Assignment of interventions (for controlled trials)

Allocation:

Sequence generation	16a	Method of generating the allocation sequence (eg, computer-generated random numbers), and list of any factors for stratification. To reduce predictability of a random sequence, details of any planned restriction (eg, blocking) should be provided in a separate document that is unavailable to those who enrol participants or assign interventions	10
Allocation concealment mechanism	16b	Mechanism of implementing the allocation sequence (eg, central telephone; sequentially numbered, opaque, sealed envelopes), describing any steps to conceal the sequence until interventions are assigned	10
Implementation	16c	Who will generate the allocation sequence, who will enrol participants, and who will assign participants to interventions	10
Blinding (masking)	17a	Who will be blinded after assignment to interventions (eg, trial participants, care providers, outcome assessors, data analysts), and how	10
	17b	If blinded, circumstances under which unblinding is permissible, and procedure for revealing a participant’s allocated intervention during the trial	10

Methods: Data collection, management, and analysis

Data collection methods	18a	Plans for assessment and collection of outcome, baseline, and other trial data, including any related processes to promote data quality (eg, duplicate measurements, training of assessors) and a description of study instruments (eg, questionnaires, laboratory tests) along with their reliability and validity, if known. Reference to where data collection forms can be found, if not in the protocol	12, Table 1
	18b	Plans to promote participant retention and complete follow-up, including list of any outcome data to be collected for participants who discontinue or deviate from intervention protocols	8,11-12,16-17
Data management	19	Plans for data entry, coding, security, and storage, including any related processes to promote data quality (eg, double data entry; range checks for data values). Reference to where details of data management procedures can be found, if not in the protocol	12,15-16
Statistical methods	20a	Statistical methods for analysing primary and secondary outcomes. Reference to where other details of the statistical analysis plan can be found, if not in the protocol	14-15
	20b	Methods for any additional analyses (eg, subgroup and adjusted analyses)	15
	20c	Definition of analysis population relating to protocol non-adherence (eg, as randomised analysis), and any statistical methods to handle missing data (eg, multiple imputation)	14-15
**Methods: Monitoring**			
Data monitoring	21a	Composition of data monitoring committee (DMC); summary of its role and reporting structure; statement of whether it is independent from the sponsor and competing interests; and reference to where further details about its charter can be found, if not in the protocol. Alternatively, an explanation of why a DMC is not needed	19
	21b	Description of any interim analyses and stopping guidelines, including who will have access to these interim results and make the final decision to terminate the trial	6,20
Harms	22	Plans for collecting, assessing, reporting, and managing solicited and spontaneously reported adverse events and other unintended effects of trial interventions or trial conduct	12
Auditing	23	Frequency and procedures for auditing trial conduct, if any, and whether the process will be independent from investigators and the sponsor	19
**Ethics and dissemination**			

For peer review only - http://bmjopen.bmj.com/site/about/guidelines.xhtml

Research ethics approval	24	Plans for seeking research ethics committee/institutional review board (REC/IRB) approval	18
Protocol amendments	25	Plans for communicating important protocol modifications (eg, changes to eligibility criteria, outcomes, analyses) to relevant parties (eg, investigators, REC/IRBs, trial participants, trial registries, journals, regulators)	19
Consent or assent	26a	Who will obtain informed consent or assent from potential trial participants or authorised surrogates, and how (see Item 32)	8
	26b	Additional consent provisions for collection and use of participant data and biological specimens in ancillary studies, if applicable	8
Confidentiality	27	How personal information about potential and enrolled participants will be collected, shared, and maintained in order to protect confidentiality before, during, and after the trial	8,12,15,16
Declaration of interests	28	Financial and other competing interests for principal investigators for the overall trial and each study site	22
Access to data	29	Statement of who will have access to the final trial dataset, and disclosure of contractual agreements that limit such access for investigators	16
Ancillary and post-trial care	30	Provisions, if any, for ancillary and post-trial care, and for compensation to those who suffer harm from trial participation	N/A
Dissemination policy	31a	Plans for investigators and sponsor to communicate trial results to participants, healthcare professionals, the public, and other relevant groups (eg, via publication, reporting in results databases, or other data sharing arrangements), including any publication restrictions	20
	31b	Authorship eligibility guidelines and any intended use of professional writers	N/A
	31c	Plans, if any, for granting public access to the full protocol, participant-level dataset, and statistical code	16
**Appendices**			
Informed consent materials	32	Model consent form and other related documentation given to participants and authorised surrogates	Upon request gap-study@bristol.ac.uk

For peer review only - http://bmjopen.bmj.com/site/about/guidelines.xhtml

Biological specimens	33	Plans for collection, laboratory evaluation, and storage of biological specimens for genetic or molecular analysis in the current trial and for future use in ancillary studies, if applicable	N/A

*It is strongly recommended that this checklist be read in conjunction with the SPIRIT 2013 Explanation & Elaboration for important clarification on the items. Amendments to the protocol should be tracked and dated. The SPIRIT checklist is copyrighted by the SPIRIT Group under the Creative Commons "Attribution-NonCommercial-NoDerivs 3.0 Unported" license.

For peer review only
